# Supplementary material for: Extensive modulation of the circulating blood proteome by hormonal contraceptive use across two population studies
Source: Commun Med (Lond). 2025 Apr 22;5:131. doi: 10.1038/s43856-025-00856-0 (PMC12015301; doi:10.1038/s43856-025-00856-0)
Supplement: Supplementary file 2 — Supplemetary Information [file 43856_2025_856_MOESM2_ESM.pdf]

Supplementary Information for: *Extensive Modulation of the Circulating Blood Proteome by Hormonal Contraceptive Use Across Two Population Studies*

Contents

Extended Material and Methods **2**

Study Sample Characteristics and General Data Overview **3**

Protein Coverage and Variation in the CHRIS Cohort **3**

Plasma Proteome Associations to Sex, Age and BMI **7**
Sex-associated plasma proteins 7
Age-associated plasma proteins 8
Body mass index associated plasma proteins 10
Proteins significantly associated with fasting status 11
Sensitivity analysis 12

Influence of Medication on the Plasma Proteome **13**
ATC level 3 Medications 13
ATC level 4 medications 19

Oral Hormonal Contraceptives Shape the Plasma Proteome in Female Study Participants **26**

Hormonal Contraceptive Use Induces Similar Proteomics Changes in an Independent Cohort **26**

Combined Hormonal Contraceptives Containing Ethinylestradiol Have a Stronger Effect Than Those with Natural Estrogens **27**

No Long-Lasting Effects of Hormonal Contraceptives on the Plasma Proteome Observed **29**

Extended Material and Methods

Table S1: Set up for gradient separation (left) and wash and equilibration (right).

Gradient pump				Regeneration pump			
Time	%A	%B	FR [ml/mn]	Time	%A	%B	FR [ml/mn]
0	97	3	0.8	0	97	3	0.8
0.7	20	80	1	5	65	35	0.8
1.5	20	80	1	5.05	97	3	1
2	97	3	1	5.75	97	3	1
5	97	3	1	5.8	97	3	0.8
5.2	97	3	0.8				

Table S2: Demographic characteristics of the BASE-II study participants included in the analysis.

	Women	Men
**n**	240	197
**Age, mean (SD)**	28.7 (3.1)	29.2 (3.0)
**Classification according to BMI, n (%)**		
**1: underweight**	22 (9.2%)	8 (4.1%)
**2: normal**	172 (71.7%)	127 (64.5%)
**3: overweight**	31 (12.9%)	53 (27.0%)
**4: obese**	15 (6.3%)	9 (4.6%)

Study Sample Characteristics and General Data Overview

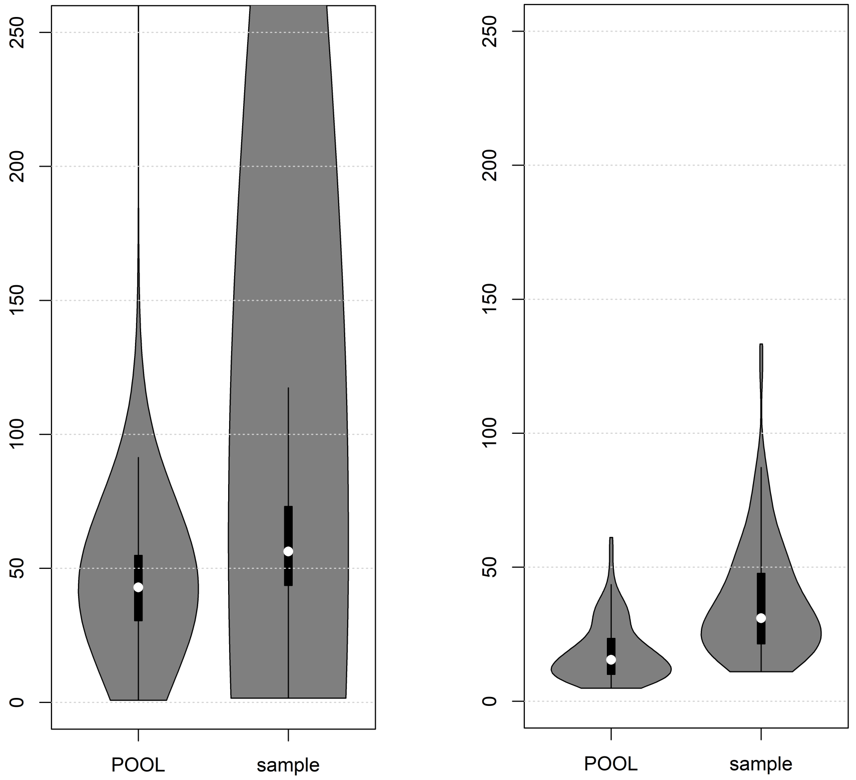

Figure S1: Distribution of coefficients of variation (CV, in %) before and after data pre-processing. Left: CVs of raw peptide data (DIA-NN normalized data). Right: CVs of protein data after normalization

Protein Coverage and Variation in the CHRIS Cohort

Table S3: Proteins with lowest coefficient of variation across study samples. CV_{study}: coefficient of variation in study samples, CV_{QC}: coefficient of variation in pooled QC samples and CV_{rel}: relative coefficient of variation expressed as the ratio between the CV in study and in pooled QC samples.

UniProt	*Gene*	*Description*	CV_{study}	CV_{QC}	CV_{rel}
P10909;P10909-2;P10909-4;P10909-5	CLU	Clusterin	11	6.27	1.75
P00734	F2	Prothrombin	13	7.02	1.85
P02768	ALB	Serum albumin	13.3	9.47	1.4
P01008	SERPINC1	Antithrombin-III	13.7	7.37	1.86
P02790	HPX	Hemopexin	13.8	7.89	1.74
P01024	C3	Complement C3	13.8	4.87	2.84
P19827	ITIH1	Inter-alpha-trypsin inhibitor heavy chain H1	13.9	6.75	2.05
P19823	ITIH2	Inter-alpha-trypsin inhibitor heavy chain H2	13.9	8.58	1.62
P02760	AMBP	Protein AMBP	14.7	8.8	1.67
P01031	C5	Complement C5	14.7	8.02	1.83
P01042;P01042-2	KNG1	Kininogen-1	14.8	8.42	1.76
Q14624;Q14624-2	ITIH4	Inter-alpha-trypsin inhibitor heavy chain H4	14.9	7.54	1.97
P04217	A1BG	Alpha-1B-glycoprotein	15.3	9.36	1.64
P02652	APOA2	Apolipoprotein A-II	15.5	8.17	1.9

UniProt	*Gene*	*Description*	CV_{study}	CV_{QC}	CV_{rel}
P00747	PLG	Plasminogen	15.6	6.52	2.39
P08603	CFH	Complement factor H	15.7	7.93	1.98
P02774;P02774-3	GC	Vitamin D-binding protein	16.3	6.13	2.66
P00751	CFB	Complement factor B	17	7.5	2.26
P09871	C1S	Complement C1s subcomponent	17.5	11.6	1.51
P02787	TF	Serotransferrin	17.8	7.59	2.35
P04217;P04217-2	A1BG	Alpha-1B-glycoprotein	18.2	10.6	1.72
P08697	SERPINF2	Alpha-2-antiplasmin	18.3	12.3	1.49
P04004	VTN	Vitronectin	18.4	8.55	2.15
P25311	AZGP1	Zinc-alpha-2-glycoprotein	18.9	9.66	1.95
P02765	AHSG	Alpha-2-HS-glycoprotein	19.1	9.31	2.05
P07357	C8A	Complement component C8 alpha chain	19.3	12.2	1.58
P01011	SERPINA3	Alpha-1-antichymotrypsin	19.5	11	1.77
P05156	CFI	Complement factor I	19.5	11.3	1.73
P00736	C1R	Complement C1r subcomponent	19.9	15.1	1.32
P06396;P06396-2	GSN	Gelsolin	20	10.5	1.91

Table S4: Proteins with the highest (relative) coefficient of variation across study samples. CV_{study}: coefficient of variation in study samples, CV_{QC}: coefficient of variation in pooled QC samples and CV_{rel}: relative coefficient of variation expressed as the ratio between the CV in study and in pooled QC samples.

UniProt	*Gene*	*Description*	CV_{study}	CV_{QC}	CV_{rel}
P01861	IGHG4	Immunoglobulin heavy constant gamma 4	89.2	12.5	7.15
P01019	AGT	Angiotensinogen	68.8	10.6	6.5
P01871	IGHM	Immunoglobulin heavy constant mu	66.7	11	6.09
A0A0B4J1U7	IGHV6-1	Immunoglobulin heavy variable 6-1	80.4	15.2	5.29
P01023	A2M	Alpha-2-macroglobulin	29.9	7.06	4.23
P06727	APOA4	Apolipoprotein A-IV	29.8	7.04	4.23
A0A0J9YXX1	IGHV5-10-1	Immunoglobulin heavy variable 5-10-1	79.5	19.2	4.13
P01877	IGHA2	Immunoglobulin heavy constant alpha 2	46.1	11.8	3.91
P00738	HP	Haptoglobin	38.7	10.1	3.84
P01859	IGHG2	Immunoglobulin heavy constant gamma 2	30.7	8.07	3.8
A0A075B6I0	IGLV8-61	Immunoglobulin lambda variable 8-61	58.7	15.6	3.76
P02656	APOC3	Apolipoprotein C-III	33.3	9.07	3.67
P04278	SHBG	Sex hormone-binding globulin	123	34.7	3.55
P01591	JCHAIN	Immunoglobulin J chain	56.7	16	3.54
P00739	HPR	Haptoglobin-related protein	41.1	11.9	3.45
P01876	IGHA1	Immunoglobulin heavy constant alpha 1	44.5	13.1	3.39
P05155;P05155-3	SERPING1	Plasma protease C1 inhibitor	55.7	16.5	3.38
P00450	CP	Ceruloplasmin	26.9	8.33	3.23
P68871	HBB	Hemoglobin subunit beta	54.5	17.1	3.18
P05546	SERPIND1	Heparin cofactor 2	25	7.99	3.13
P19652	ORM2	Alpha-1-acid glycoprotein 2	31.3	10	3.12
O43866	CD5L	CD5 antigen-like	54.4	17.8	3.06
P02748	C9	Complement component C9	27.7	9.33	2.96
A0A0B4J1V2	IGHV2-26	Immunoglobulin heavy variable 2-26	46.8	15.9	2.94
P02750	LRG1	Leucine-rich alpha-2-glycoprotein	31.3	10.8	2.89
P01834	IGKC	Immunoglobulin kappa constant	22	7.62	2.89
P04114	APOB	Apolipoprotein B-100	28.6	9.9	2.89
P01024	C3	Complement C3	13.8	4.87	2.84
P08185	SERPINA6	Corticosteroid-binding globulin	48.3	17.1	2.83
P01602	IGKV1-5	Immunoglobulin kappa variable 1-5	41.1	14.6	2.82

Table S5: Common disease biomarker proteins quantified in the present data set. CV_{study}: coefficient of variation in study samples and CV_{QC}: coefficient of variation in pooled QC samples.

UniProt	*Genes*	*Description*	CV_{study}	CV_{QC}
P02768	ALB	Serum albumin	13.3	9.47
P02763	ORM1	Alpha-1-acid glycoprotein 1	30	11.6
P01009	SERPINA1	Alpha-1-antitrypsin	21.4	7.95
P08697	SERPINF2	Alpha-2-antiplasmin	18.3	12.3
P02765	AHSG	Alpha-2-HS-glycoprotein	19.1	9.31
P01023	A2M	Alpha-2-macroglobulin	29.9	7.06
P01008	SERPINC1	Antithrombin-III	13.7	7.37
P04114	APOB	Apolipoprotein B-100	28.6	9.9
P00450	CP	Ceruloplasmin	26.9	8.33
P06276	BCHE	Cholinesterase	37	34.8
P09871	C1S	Complement C1s subcomponent	17.5	11.6
P02745	C1QA	Complement C1q subcomponent subunit A	31.1	24
P01024	C3	Complement C3	13.8	4.87
P0C0L4	C4A	Complement C4-A	46.3	18.8
P01031	C5	Complement C5	14.7	8.02
P00740	F9	Coagulation factor IX	47.2	30.1
P00742	F10	Coagulation factor X	21.5	16.9
P00488	F13A1	Coagulation factor XIII A chain	43.7	30.2
P05160	F13B	Coagulation factor XIII B chain	54.4	30.8
P02671	FGA	Fibrinogen alpha chain	71.2	31.1
P02675	FGB	Fibrinogen beta chain	65.2	29.2
P02751	FN1	Fibronectin	88	61.1
P00738	HP	Haptoglobin	38.7	10.1
P02790	HPX	Hemopexin	13.8	7.89
P08519	LPA	Apolipoprotein(a)	134	56.5
P00747	PLG	Plasminogen	15.6	6.52
P02766	TTR	Transthyretin	43	20
P07225	PROS1	Vitamin K-dependent protein S	20.6	14.4
P02753	RBP4	Retinol-binding protein 4	23.9	9.48
P04278	SHBG	Sex hormone-binding globulin	123	34.7
P05543	SERPINA7	Thyroxine-binding globulin	35.9	18.3

Table S6: Correlation between clinical laboratory measurements and quantified protein abundances.

Laboratory.parameter	UniProt.ID	HGNC.symbol	rho	p.value
Antithrombin (%)	P01008	SERPINC1	0.366	1.23e-110
Albumin (g/dL)	P02768	ALB	0.32	1.57e-83
HDL (mg/dL)	P02647	APOA1	0.408	1.26e-139
LDL (mg/dL)	P04114	APOB	0.787	0
Triglycerides (mg/dL)	P02656	APOC3	0.596	0
Transferrin (mg/dL)	P02787	TF	0.686	0
HGN (h/dL)	P69905	HBA1	0.48	8.2e-200

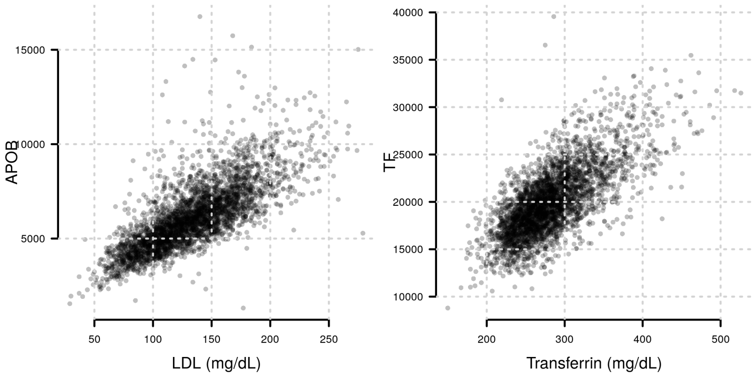

Figure S2: Correlation between quantified protein abundances and related diagnostic assays. Shown are MS-determind, relative protein abundances (normalized intensities) on the y-axis and related diagnostic assays on the x-axis.

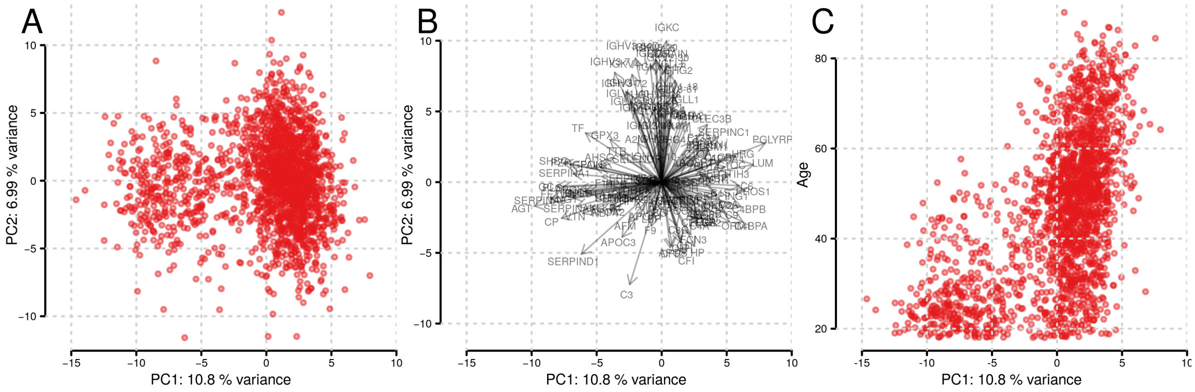

Figure S3: Principal Component Analysis (PCA) of the plasma proteome of female CHRIS participants. A) Sample grouping by PC1 and PC2. Each point represents an individual with a different color used for men (blue) and women (red). B) Loadings from the PCA. Each arrow represents one protein with its length and direction indicating their impact and importance for that principal component. C) Relationship between PC1 (x-axis) and the participant's age (y-axis). Points are colored by participants' sex.

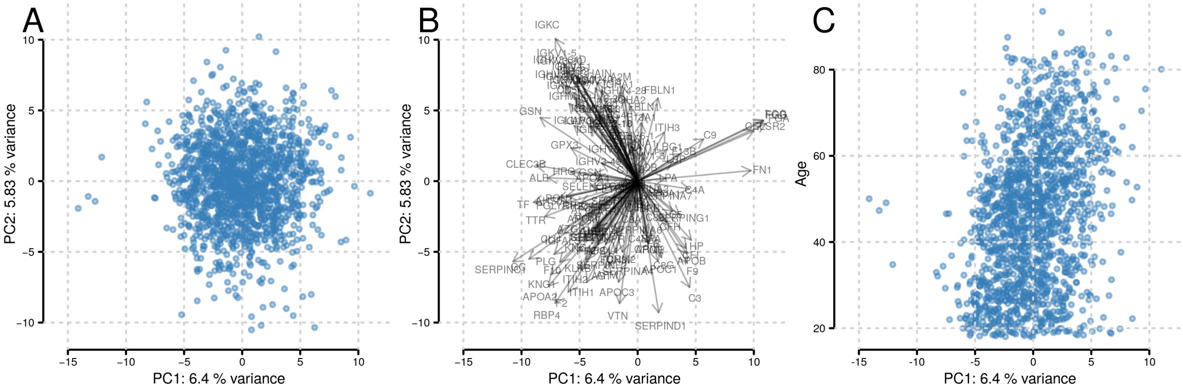

Figure S4: Principal Component Analysis (PCA) of the plasma proteome of male CHRIS participants. A) Sample grouping by PC1 and PC2. Each point represents an individual with a different color used for men (blue) and women (red). B) Loadings from the PCA. Each arrow represents one protein with its length and direction indicating their impact and importance for that principal component. C) Relationship between PC1 (x-axis) and the participant's age (y-axis). Points are colored by participants' sex.

Table S7: Variables significantly associated with principal component 1. Results are ordered by p-value.

Variable	*Description*	*coef*	*p − value*
G03A	Hormonal contraceptives for systemic use	-6.54	5.67e-299
Age_10	Participant's age	0.561	1.13e-72
G03H	Antiandrogens	-8.72	1.11e-48
Sex	Sex	-0.974	1.53e-27
G02B	Contraceptives for topical use	-3.14	3.55e-19
BMI4	BMI category 4 (obese)	0.419	0.000686
G03F	Progestogens and estrogens in combination	-1.77	0.00135

Plasma Proteome Associations to Sex, Age and BMI

Sex-associated plasma proteins

Table S8: Proteins with significant difference in abundance between female and male study participants. $coef$, ES and p_{adj}: coefficient (representing the differential abundance in log2 scale), effect size and p-value adjusted for multiple hypothesis testing. Proteins are ordered by p-value.

UniProt	*Gene*	*Description*	*coef*	p_{adj}	*ES*
P02753	RBP4	Retinol-binding protein 4	-0.306	3.11e-152	-0.908
P00450	CP	Ceruloplasmin	0.231	2.93e-120	0.668
P80108	GPLD1	Phosphatidylinositol-glycan-specific phospholipase D	-0.306	3.14e-90	-0.709
P36955	SERPINF1	Pigment epithelium-derived factor	-0.202	2.51e-83	-0.671
P02766	TTR	Transthyretin	-0.387	1.28e-81	-0.676
P25311	AZGP1	Zinc-alpha-2-glycoprotein	-0.177	4e-80	-0.662
P69905	HBA1	Hemoglobin subunit alpha	-0.347	6.89e-78	-0.667
P02750	LRG1	Leucine-rich alpha-2-glycoprotein	0.281	3.39e-77	0.647
P68871	HBB	Hemoglobin subunit beta	-0.313	1.92e-74	-0.654
O75636	FCN3	Ficolin-3	-0.368	3.52e-70	-0.616
P04278	SHBG	Sex hormone-binding globulin	0.633	5.19e-69	0.512
P13671	C6	Complement component C6	0.199	3.2e-68	0.615
P02748	C9	Complement component C9	0.199	4.41e-45	0.504
P05090	APOD	Apolipoprotein D	-0.191	1.14e-42	-0.474
P01871	IGHM	Immunoglobulin heavy constant mu	0.331	2.25e-33	0.436

UniProt	*Gene*	*Description*	*coef*	p_{adj}	*ES*
P01023	A2M	Alpha-2-macroglobulin	0.165	2.13e-29	0.398
O43866	CD5L	CD5 antigen-like	0.286	2.24e-25	0.382
P01861	IGHG4	Immunoglobulin heavy constant gamma 4	-0.43	1.88e-22	-0.37
P01877	IGHA2	Immunoglobulin heavy constant alpha 2	-0.209	2.22e-17	-0.324
P01876	IGHA1	Immunoglobulin heavy constant alpha 1	-0.198	7.73e-15	-0.3
P00739	HPR	Haptoglobin-related protein	-0.177	5.15e-14	-0.293
P06727	APOA4	Apolipoprotein A-IV	-0.117	3.34e-13	-0.272

Age-associated plasma proteins

Table S9: Proteins significantly associated with participants' age. *coef*, *ES* and p_{adj}: coefficient (representing the log2 change in abundance in 10 years), effect size (for 10-year change) and p-value adjusted for multiple hypothesis testing. Proteins are ordered by p-value.

UniProt	*Gene*	*Description*	*coef*	p_{adj}	*ES*
P35858;P35858-2	IGFALS	Insulin-like growth factor-binding protein complex acid labile subunit	-0.11	4.3e-137	-0.26
P04004	VTN	Vitronectin	-0.057	2.8e-111	-0.22
P02787	TF	Serotransferrin	-0.045	6.9e-63	-0.18
P02765	AHSG	Alpha-2-HS-glycoprotein	-0.05	7.4e-60	-0.18
P51884	LUM	Lumican	0.063	9.8e-55	0.16
P01031	C5	Complement C5	0.036	2.2e-50	0.17
P04114	APOB	Apolipoprotein B-100	0.063	1.9e-43	0.16
O14791;O14791-2;O14791-3	APOL1	Apolipoprotein L1	-0.068	4.7e-43	-0.15
P00734	F2	Prothrombin	-0.031	1.9e-42	-0.16
Q06033;Q06033-2	ITIH3	Inter-alpha-trypsin inhibitor heavy chain H3	0.085	6.7e-42	0.15
P01619	IGKV3-20	Immunoglobulin kappa variable 3-20	-0.082	7.4e-41	-0.15
P01008	SERPINC1	Antithrombin-III	-0.027	1.2e-40	-0.14
P02774;P02774-3	GC	Vitamin D-binding protein	-0.028	7.7e-40	-0.13
P04003	C4BPA	C4b-binding protein alpha chain	0.042	1.7e-38	0.14
P02750	LRG1	Leucine-rich alpha-2-glycoprotein	0.06	1.1e-36	0.14
P08697	SERPINF2	Alpha-2-antiplasmin	-0.039	5.3e-35	-0.14
P00742	F10	Coagulation factor X	-0.044	1.1e-33	-0.14
P04217	A1BG	Alpha-1B-glycoprotein	0.031	1e-32	0.13
P01602	IGKV1-5	Immunoglobulin kappa variable 1-5	-0.073	3.7e-32	-0.14
P02748	C9	Complement component C9	0.049	6.2e-28	0.12
P00747	PLG	Plasminogen	-0.026	1.1e-27	-0.12
P01703	IGLV1-40	Immunoglobulin lambda variable 1-40	-0.11	8.4e-27	-0.13
P01780	IGHV3-7	Immunoglobulin heavy variable 3-7	-0.078	2.7e-26	-0.12
P00746	CFD	Complement factor D	0.068	3.1e-25	0.12
P06312	IGKV4-1	Immunoglobulin kappa variable 4-1	-0.069	3.1e-24	-0.12
P01871	IGHM	Immunoglobulin heavy constant mu	-0.086	4.5e-23	-0.11
P02760	AMBP	Protein AMBP	0.025	2.7e-22	0.11
O43866	CD5L	CD5 antigen-like	-0.083	4e-22	-0.11
P06310	IGKV2-30	Immunoglobulin kappa variable 2-30	-0.065	6.9e-22	-0.11
P27169	PON1	Serum paraoxonase/arylesterase 1	-0.046	5e-19	-0.1
P02768	ALB	Serum albumin	-0.02	9.8e-19	-0.1
P19827	ITIH1	Inter-alpha-trypsin inhibitor heavy chain H1	-0.021	6.5e-17	-0.1
P08185	SERPINA6	Corticosteroid-binding globulin	-0.046	4.8e-16	-0.082
P20851;P20851-2	C4BPB	C4b-binding protein beta chain	0.044	7.9e-16	0.092
A0A0J9YX35	IGHV3-64D	Immunoglobulin heavy variable 3-64D	-0.05	2e-15	-0.095
P05452	CLEC3B	Tetranectin	-0.029	2.5e-15	-0.092

UniProt	*Gene*	*Description*	*coef*	p_{adj}	*ES*
P04217;P04217-2	A1BG	Alpha-1B-glycoprotein	0.024	4.8e-15	0.092
P01877	IGHA2	Immunoglobulin heavy constant alpha 2	0.061	5.2e-15	0.094
P00748	F12	Coagulation factor XII	-0.043	7.9e-15	-0.093
P02790	HPX	Hemopexin	0.018	4.1e-14	0.09
P80748	IGLV3-21	Immunoglobulin lambda variable 3-21	-0.073	5.7e-14	-0.092
P04196	HRG	Histidine-rich glycoprotein	0.032	2.1e-13	0.082
P01011	SERPINA3	Alpha-1-antichymotrypsin	0.025	2.8e-13	0.088
P00738	HP	Haptoglobin	0.056	3.7e-13	0.088
P01705	IGLV2-23	Immunoglobulin lambda variable 2-23	-0.074	5.4e-13	-0.089
P02766	TTR	Transthyretin	-0.047	9.8e-13	-0.083
P10643	C7	Complement component C7	0.035	2.1e-12	0.085
P01019	AGT	Angiotensinogen	-0.041	2.1e-12	-0.061
P06727	APOA4	Apolipoprotein A-IV	0.035	5.6e-12	0.081
P01876	IGHA1	Immunoglobulin heavy constant alpha 1	0.055	2e-11	0.083
P02749	APOH	Beta-2-glycoprotein 1	0.034	3.9e-11	0.081
Q96PD5	PGLYRP2	N-acetylmuramoyl-L-alanine amidase	0.026	4.8e-11	0.068
Q9UGM5	FETUB	Fetuin-B	-0.062	5.7e-11	-0.071
P01042;P01042-2	KNG1	Kininogen-1	-0.015	5.2e-10	-0.073
P01599	IGKV1-17	Immunoglobulin kappa variable 1-17	-0.06	1.3e-09	-0.077
P05090	APOD	Apolipoprotein D	0.028	2.2e-09	0.07
A0A0C4DH31	IGHV1-18	Immunoglobulin heavy variable 1-18	-0.058	2.4e-08	-0.072
A0A075B6I0	IGLV8-61	Immunoglobulin lambda variable 8-61	-0.056	4.6e-08	-0.071
P02656	APOC3	Apolipoprotein C-III	0.031	4.8e-08	0.07
P05156	CFI	Complement factor I	0.019	1.1e-07	0.066
P08603	CFH	Complement factor H	0.014	1.9e-07	0.063
P22352	GPX3	Glutathione peroxidase 3	-0.031	8.9e-07	-0.063
P03952	KLKB1	Plasma kallikrein	-0.02	1.6e-06	-0.064
P23142;P23142-4	FBLN1	Fibulin-1	0.037	2.2e-06	0.063
P01042-2	KNG1	Isoform LMW of Kininogen-1	-0.024	6.3e-06	-0.059
A0A075B6J9	IGLV2-18	Immunoglobulin lambda variable 2-18	-0.061	8.5e-06	-0.062
P25311	AZGP1	Zinc-alpha-2-glycoprotein	0.015	1.1e-05	0.057
A0A0B4J1V2	IGHV2-26	Immunoglobulin heavy variable 2-26	-0.042	1.3e-05	-0.061
A0A0B4J1Y9	IGHV3-72	Immunoglobulin heavy variable 3-72	-0.051	1.3e-05	-0.06
P01701	IGLV1-51	Immunoglobulin lambda variable 1-51	-0.043	0.00017	-0.055
P80108	GPLD1	Phosphatidylinositol-glycan-specific phospholipase D	-0.021	0.00044	-0.049
P09871	C1S	Complement C1s subcomponent	0.014	0.00053	0.052
P0C0L4	C4A	Complement C4-A	0.037	0.00064	0.052
A0A0A0MS15	IGHV3-49	Immunoglobulin heavy variable 3-49	-0.029	0.00068	-0.052
P02679;P02679-2	FGG	Fibrinogen gamma chain	0.039	0.00068	0.052
P19652	ORM2	Alpha-1-acid glycoprotein 2	-0.021	0.0013	-0.048
Q16610;Q16610-4	ECM1	Extracellular matrix protein 1	-0.024	0.0017	-0.049
P02652	APOA2	Apolipoprotein A-II	-0.011	0.0017	-0.048
P23142	FBLN1	Fibulin-1	0.033	0.0024	0.048
P02675	FGB	Fibrinogen beta chain	0.039	0.0025	0.049
Q9HCU4	CELSR2	Cadherin EGF LAG seven-pass G-type receptor 2	0.054	0.0031	0.048
P01857	IGHG1	Immunoglobulin heavy constant gamma 1	-0.017	0.0039	-0.047
P43652	AFM	Afamin	-0.014	0.0051	-0.045
P02649	APOE	Apolipoprotein E	0.022	0.0077	0.046
P01023	A2M	Alpha-2-macroglobulin	-0.018	0.009	-0.042
P07225	PROS1	Vitamin K-dependent protein S	0.013	0.011	0.041
P10909;P10909-2;P10909-4;P10909-5	CLU	Clusterin	-0.007	0.012	-0.044
P05543	SERPINA7	Thyroxine-binding globulin	-0.02	0.017	-0.039
P00450	CP	Ceruloplasmin	0.011	0.023	0.032
O75882-2	ATRN	Isoform 2 of Attractin	0.014	0.027	0.042
P02671	FGA	Fibrinogen alpha chain	0.034	0.049	0.041

The age association for the 2 most significant proteins is shown below.

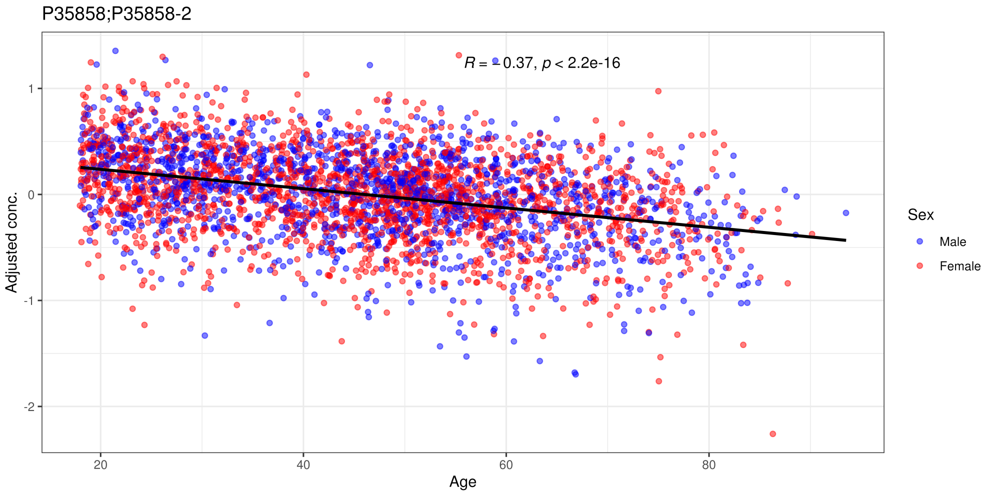

Figure S5: Age dependency of the protein IGFALS. Shown are sex, BMI, HCU and fasting status adjusted abundances against participants' age. The solid black line represents the linear regression line.

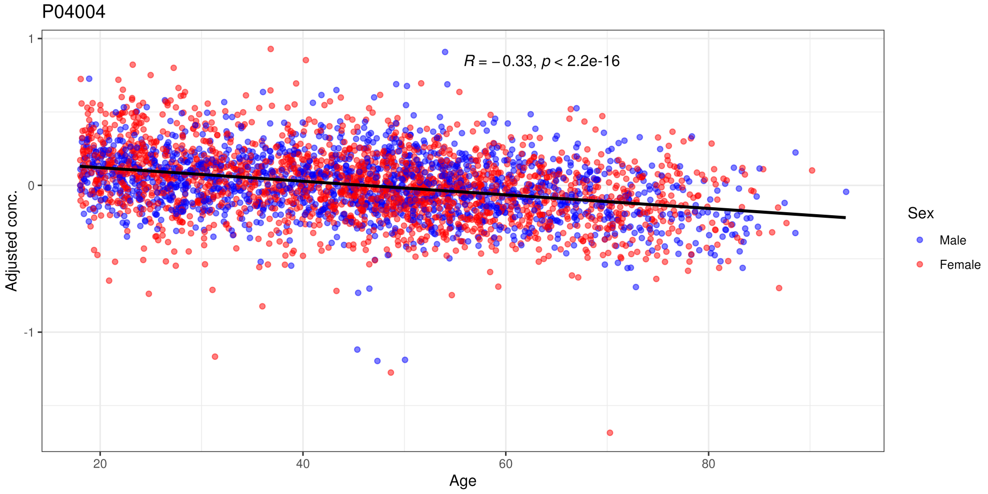

Figure S6: Age dependency of the protein VTN. Shown are sex, BMI, HCU and fasting status adjusted abundances against participants' age. The solid black line represents the linear regression line.

Body mass index associated plasma proteins

Proteins with significant differences in concentrations between body mass index (BMI) categories 1 (BMI < 18.5), 3 (25 <= BMI < 30) and 4 (BMI > 30) to the *normal* category 2 (18.5 <= BMI < 25) are listed in the tables below. Proteins with an adjusted p-value smaller than 0.05 and a difference in concentrations larger than the coefficient of variation in QC (study pool) samples are considered significant.

Table S10: Proteins with significant difference in abundance between participants with a BMI < 18.5 (BMI1) and participants with a BMI between 18.5 and 25 (BMI2; normal). $coef$, ES and p_{adj}: coefficient (representing the differential abundance in log2 scale), effect size and p-value adjusted for multiple hypothesis testing. Proteins are ordered by p-value.

UniProt	*Gene*	*Description*	$coef$	p_{adj}	ES
P08603	CFH	Complement factor H	-0.132	0.00215	-0.578
P01023	A2M	Alpha-2-macroglobulin	0.237	0.00405	0.571
P02763	ORM1	Alpha-1-acid glycoprotein 1	-0.218	0.0323	-0.511
P01024	C3	Complement C3	-0.0961	0.0418	-0.481

Table S11: Proteins with significant difference in abundance between participants with a BMI between 25 and 30 (BMI3) and participants with a BMI between 18.5 and 25 (BMI2; normal). $coef$, ES and p_{adj}: coefficient (representing the differential abundance in log2 scale) effect size and p-value adjusted for multiple hypothesis testing. Proteins are ordered by p-value.

UniProt	*Gene*	*Description*	$coef$	p_{adj}	ES
P01024	C3	Complement C3	0.112	6.38e-52	0.561
P01023	A2M	Alpha-2-macroglobulin	-0.199	3.42e-36	-0.48
P05090	APOD	Apolipoprotein D	-0.181	2.55e-32	-0.448
P06727	APOA4	Apolipoprotein A-IV	-0.12	1.47e-11	-0.277

Table S12: Proteins with significant difference in abundance between participants with a BMI > 30 (BMI4) and participants with a BMI between 18.5 and 25 (BMI2; normal). $coef$, ES and p_{adj}: coefficient (representing the differential abundance in log2 scale) effect size and p-value adjusted for multiple hypothesis testing. Proteins are ordered by p-value.

UniProt	*Gene*	*Description*	$coef$	p_{adj}	ES
P01024	C3	Complement C3	0.234	1.62e-128	1.17
P05090	APOD	Apolipoprotein D	-0.401	1.16e-91	-0.992
P08603	CFH	Complement factor H	0.213	7.13e-83	0.933
P01008	SERPINC1	Antithrombin-III	-0.151	6.26e-61	-0.755
P43652	AFM	Afamin	0.247	7.59e-56	0.8
P04004	VTN	Vitronectin	0.179	4.34e-55	0.698
P05156	CFI	Complement factor I	0.219	6.13e-52	0.761
P00751	CFB	Complement factor B	0.167	7.25e-41	0.692
O95445	APOM	Apolipoprotein M	-0.23	2.04e-36	-0.651
P02774;P02774-3	GC	Vitamin D-binding protein	-0.112	5.04e-32	-0.515
P01023	A2M	Alpha-2-macroglobulin	-0.242	8.25e-32	-0.583
P25311	AZGP1	Zinc-alpha-2-glycoprotein	-0.151	3.62e-30	-0.564
P04278	SHBG	Sex hormone-binding globulin	-0.577	2.09e-29	-0.466
P06727	APOA4	Apolipoprotein A-IV	-0.207	5.29e-21	-0.48
P05546	SERPIND1	Heparin cofactor 2	0.15	3.75e-16	0.423
P02763	ORM1	Alpha-1-acid glycoprotein 1	0.176	2.67e-15	0.411
P00738	HP	Haptoglobin	0.245	1.8e-12	0.386
O43866	CD5L	CD5 antigen-like	-0.277	8.28e-12	-0.37
P19652	ORM2	Alpha-1-acid glycoprotein 2	0.141	5.59e-09	0.324
P01871	IGHM	Immunoglobulin heavy constant mu	-0.244	7.78e-09	-0.321

Proteins significantly associated with fasting status

Table S13: Proteins with significant difference in abundance between participants declared to not have fasted against those who did. $coef$, ES and p_{adj}: coefficient (representing the differential abundance in log2 scale) effect size and p-value adjusted for multiple hypothesis testing. Proteins are ordered by p-value.

UniProt	*Gene*	*Description*	$coef$	p_{adj}	ES
P06727	APOA4	Apolipoprotein A-IV	0.126	0.00158	0.291

Sensitivity analysis

To evaluate the influence of hormonal contraceptive use on results for age, sex and BMI associations, the analysis was repeated without adjustment for hormonal contraceptive use. Proteins that were found significantly associated with sex in this analysis, but were no longer significant after adjustment for hormonal contraceptive use are listed in the table below.

Table S14: Proteins significantly associated with sex in an analysis without adjusting for hormonal contraceptive use that were no longer significant after adjustment. $coef$ and p: coefficient and bonferroni adjusted p-value from the analysis without adjustment for hormonal contraceptive use. $coef_{adj}$, p_{adj}, $coef_{HCU}$ and p_{HCU}: coefficients and bonferroni adjusted p-values for sex association and hormoman contraceptive use association from the analysis with adjustment for hormonal contraceptive use.

Gene	$coef$	p	$coef_{adj}$	p_{adj}	$coef_{HCU}$	p_{HCU}
SERPINA7	0.308	5.29e-70	0.216	8.47e-36	0.583	2.56e-78
PZP	0.476	3.66e-63	0.347	9.28e-34	0.82	2.45e-57
AGT	0.35	1.89e-58	0.118	3.76e-10	1.47	0
FETUB	0.452	7.8e-54	0.26	1.74e-19	1.22	9.73e-125
SERPINA6	0.274	1.16e-48	0.14	3.46e-14	0.852	7.89e-153
PGLYRP2	-0.169	1.42e-38	-0.061	1e-05	-0.683	3.7e-212
SERPINA1	0.135	6.47e-37	0.0734	3.55e-11	0.392	2e-96
SERPING1	-0.314	1.17e-20	-0.176	5.86e-06	-0.874	4.95e-48

Proteins significantly associated with age from an analysis without adjustment for hormonal contraceptive use that were no longer significant after adjustment for hormonal contraceptive use are listed in the table below.

Table S15: Proteins significantly associated with age in an analysis without adjusting for hormonal contraceptive use that were no longer significant after adjustment. $coef$ and p: coefficient and bonferroni adjusted p-value from the analysis without adjustment for hormonal contraceptive use. $coef_{adj}$, p_{adj}, $coef_{HCU}$ and p_{HCU}: coefficients and bonferroni adjusted p-values for age association and hormonal contraceptive use association from the analysis with adjustment for hormonal contraceptive use.

Gene	$coef$	p	$coef_{adj}$	p_{adj}	$coef_{HCU}$	p_{HCU}
SERPINA1	-0.0299	4.79e-19	-0.00795	1	0.392	2e-96
C6	0.0293	6.8e-16	0.0113	0.143	-0.32	9.39e-55
SHBG	-0.0965	1.8e-14	0.0126	1	1.95	2.06e-185
PZP	-0.0676	1.91e-13	-0.0216	1	0.82	2.45e-57
SERPIND1	-0.0299	3.27e-13	-0.00828	1	0.385	4.97e-64
SERPING1	0.073	1.47e-11	0.024	1	-0.874	4.95e-48
C3	-0.0124	5.27e-08	-0.00522	1	0.127	1.32e-24
C1QC	0.0207	1.87e-07	0.00875	1	-0.213	6.2e-23
HPR	-0.0365	2.35e-06	-0.0207	0.323	0.281	1.03e-10
ORM1	0.0247	4.01e-06	0.0037	1	-0.375	1.26e-42
C1QB	0.0179	7.1e-05	0.00755	1	-0.184	1.7e-15
CD14	0.0321	9.31e-05	0.0139	1	-0.325	1.45e-14
C1R	0.0158	0.000111	0.00616	1	-0.172	8.53e-17

Gene	*coef*	*p*	$coef_{adj}$	p_{adj}	$coef_{HCU}$	p_{HCU}
FN1	0.0372	0.0319	0.0208	1	-0.293	0.000248
APOM	0.0135	0.0414	0.00366	1	-0.175	1.2e-12

Influence of Medication on the Plasma Proteome

ATC level 3 Medications

Associations were evaluated for 27 ATC level 3 medications taken on a regular basis (at least twice per week) by more than 14 study participants. Significant associations are shown for each medication in the tables below. For 11 medications no significant association was identified.

Table S16: Overview of association results for ATC level 3 medications in the CHRIS study subset. Only medications taken on a regular basis by more than 14 of the in total 3,632 study participants were considered. Columns *Participants* and *Proteins* list the number of participants taking the medication and number of significantly associated proteins.

ATC	Name	Participants	Proteins
G03A	HORMONAL CONTRACEPTIVES FOR SYSTEMIC USE	286	50
H03A	THYROID PREPARATIONS	262	0
B01A	ANTITHROMBOTIC AGENTS	243	2
C10A	LIPID MODIFYING AGENTS, PLAIN	203	1
C09A	ACE INHIBITORS, PLAIN	156	0
C07A	BETA BLOCKING AGENTS	149	1
N06A	ANTIDEPRESSANTS	141	0
C09D	ANGIOTENSIN II ANTAGONISTS, COMBINATIONS	113	1
A02B	DRUGS FOR PEPTIC ULCER AND GASTRO-OESOPHAGEAL REFLUX DISEASE (GORD)	94	0
C08C	SELECTIVE CALCIUM CHANNEL BLOCKERS WITH MAINLY VASCULAR EFFECTS	91	0
C09C	ANGIOTENSIN II ANTAGONISTS, PLAIN	74	1
A12A	CALCIUM	65	2
C09B	ACE INHIBITORS, COMBINATIONS	59	0
A10B	BLOOD GLUCOSE LOWERING DRUGS, EXCL. INSULINS	57	4
M01A	ANTIINFLAMMATORY AND ANTIRHEUMATIC PRODUCTS, NON-STEROIDS	48	3
G02B	CONTRACEPTIVES FOR TOPICAL USE	46	19
G04C	DRUGS USED IN BENIGN PROSTATIC HYPERTROPHY	46	4
M04A	ANTIGOUT PREPARATIONS	36	2
N03A	ANTIEPILEPTICS	34	1
N05C	HYPNOTICS AND SEDATIVES	32	0
S01E	ANTIGLAUCOMA PREPARATIONS AND MIOTICS	30	0
R03A	ADRENERGICS, INHALANTS	29	1
N05B	ANXIOLYTICS	24	0
N05A	ANTIPSYCHOTICS	21	0
G03F	PROGESTOGENS AND ESTROGENS IN COMBINATION	18	0
G03H	ANTIANDROGENS	16	38
N04B	DOPAMINERGIC AGENTS	16	1

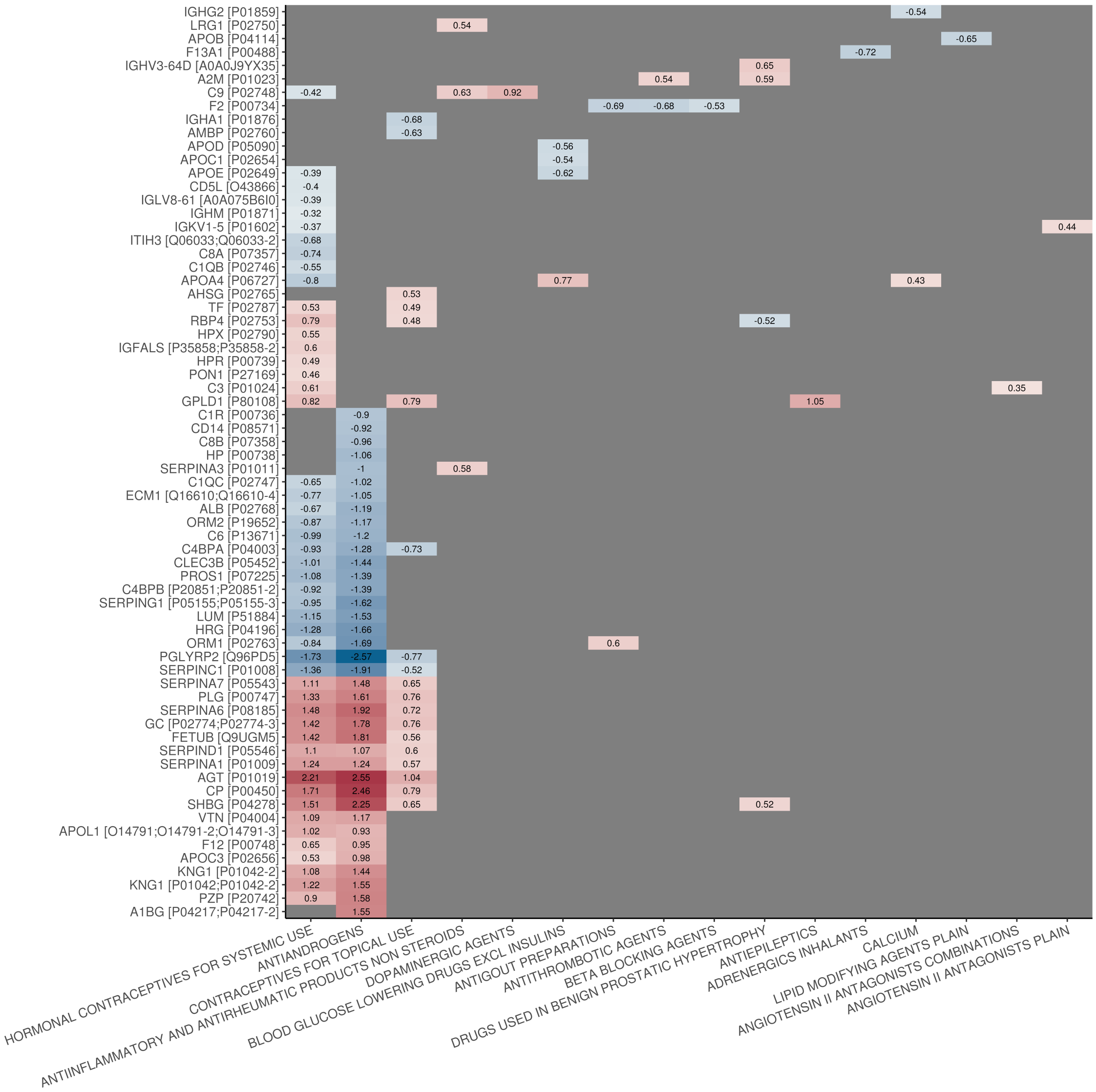

Figure S7: Significant associations between proteins (rows) and ATC level 3 medications (columns). Effect sizes (as a number and color coded) are only shown for significant associations. Ordering of rows and columns represent the result from a hierarchical cluster analysis (Ward clustering of an Euclidean dissimilarity matrix).

Table S17: Proteins significantly associated with ATC level 3 medication *HORMONAL CONTRACEPTIVES FOR SYSTEMIC USE* (ATC3 G03A). $coef$ and p_{adj}: coefficient (representing the differential abundance in log2 scale) and p-value adjusted for multiple hypothesis testing. ES effect size. Proteins are ordered by p-value.

$UniProt$	$Gene$	$Description$	$coef$	p_{adj}	ES
P01019	AGT	Angiotensinogen	1.46	0	2.21
P00450	CP	Ceruloplasmin	0.589	3.01e-206	1.71
Q96PD5	PGLYRP2	N-acetylmuramoyl-L-alanine amidase	-0.657	4.3e-179	-1.73
P04278	SHBG	Sex hormone-binding globulin	1.86	1.51e-157	1.51
P08185	SERPINA6	Corticosteroid-binding globulin	0.84	9e-135	1.48
P02774;P02774-3	GC	Vitamin D-binding protein	0.309	3.46e-128	1.42

UniProt	*Gene*	*Description*	*coef*	p_{adj}	*ES*
Q9UGM5	FETUB	Fetuin-B	1.22	1.15e-114	1.42
P01008	SERPINC1	Antithrombin-III	-0.273	4.4e-106	-1.36
P00747	PLG	Plasminogen	0.299	6.25e-98	1.33
P04196	HRG	Histidine-rich glycoprotein	-0.496	6.92e-86	-1.28
P01009	SERPINA1	Alpha-1-antitrypsin	0.383	1.4e-85	1.24
P01042;P01042-2	KNG1	Kininogen-1	0.254	1.32e-76	1.22
P51884	LUM	Lumican	-0.449	8.51e-75	-1.15
P04004	VTN	Vitronectin	0.28	2.26e-74	1.09
P05543	SERPINA7	Thyroxine-binding globulin	0.57	4.04e-68	1.11
P07225	PROS1	Vitamin K-dependent protein S	-0.333	5.97e-63	-1.08
P05546	SERPIND1	Heparin cofactor 2	0.391	8.15e-60	1.1
P01042-2	KNG1	Isoform LMW of Kininogen-1	0.438	1.21e-58	1.08
O14791;O14791-2;O14791-3	APOL1	Apolipoprotein L1	0.468	1.6e-54	1.02
P05452	CLEC3B	Tetranectin	-0.324	2.04e-51	-1.01
P13671	C6	Complement component C6	-0.321	4.84e-50	-0.992
P04003	C4BPA	C4b-binding protein alpha chain	-0.282	6.7e-49	-0.929
P05155;P05155-3	SERPING1	Plasma protease C1 inhibitor	-0.878	3.34e-44	-0.949
P20742	PZP	Pregnancy zone protein	0.752	4.72e-44	0.896
P20851;P20851-2	C4BPB	C4b-binding protein beta chain	-0.445	1.43e-43	-0.919
P19652	ORM2	Alpha-1-acid glycoprotein 2	-0.377	1.03e-36	-0.866
P02763	ORM1	Alpha-1-acid glycoprotein 1	-0.359	1.03e-35	-0.84
P80108	GPLD1	Phosphatidylinositol-glycan-specific phospholipase D	0.354	1.33e-35	0.822
P02753	RBP4	Retinol-binding protein 4	0.265	7.03e-35	0.789
P06727	APOA4	Apolipoprotein A-IV	-0.346	5.35e-33	-0.802
Q16610;Q16610-4	ECM1	Extracellular matrix protein 1	-0.38	1.91e-27	-0.765
P07357	C8A	Complement component C8 alpha chain	-0.211	1.73e-25	-0.744
Q06033;Q06033-2	ITIH3	Inter-alpha-trypsin inhibitor heavy chain H3	-0.388	2.56e-24	-0.676
P02768	ALB	Serum albumin	-0.134	2.02e-22	-0.667
P35858;P35858-2	IGFALS	Insulin-like growth factor-binding protein complex acid labile subunit	0.266	4.24e-22	0.601
P01024	C3	Complement C3	0.123	8.19e-21	0.614
P00748	F12	Coagulation factor XII	0.306	2.49e-20	0.653
P02747	C1QC	Complement C1q subcomponent subunit C	-0.208	1.24e-19	-0.65
P02787	TF	Serotransferrin	0.134	3.76e-15	0.53
P02790	HPX	Hemopexin	0.111	2.46e-14	0.547
P02746	C1QB	Complement C1q subcomponent subunit B	-0.181	1.42e-13	-0.549
P02656	APOC3	Apolipoprotein C-III	0.229	9.57e-13	0.529
P00739	HPR	Haptoglobin-related protein	0.299	5.22e-11	0.494
P27169	PON1	Serum paraoxonase/arylesterase 1	0.2	7.97e-10	0.459
P02748	C9	Complement component C9	-0.166	1.33e-08	-0.42
O43866	CD5L	CD5 antigen-like	-0.298	2.27e-07	-0.398
A0A075B6I0	IGLV8-61	Immunoglobulin lambda variable 8-61	-0.31	1.11e-06	-0.395
P02649	APOE	Apolipoprotein E	-0.187	1.62e-06	-0.388
P01602	IGKV1-5	Immunoglobulin kappa variable 1-5	-0.196	6.85e-06	-0.365
P01871	IGHM	Immunoglobulin heavy constant mu	-0.245	0.000116	-0.323

Table S18: Proteins significantly associated with ATC level 3 medication *ANTIANDROGENS* (ATC3 G03H). *coef* and p_{adj}: coefficient (representing the differential abundance in log2 scale) and p-value adjusted for multiple hypothesis testing. *ES* effect size. Proteins are ordered by p-value.

UniProt	*Gene*	*Description*	*coef*	p_{adj}	*ES*
P01019	AGT	Angiotensinogen	1.68	5.77e-43	2.55
P00450	CP	Ceruloplasmin	0.85	9.01e-36	2.46
Q96PD5	PGLYRP2	N-acetylmuramoyl-L-alanine amidase	-0.972	4.27e-32	-2.57
P04278	SHBG	Sex hormone-binding globulin	2.78	2.76e-28	2.25
P08185	SERPINA6	Corticosteroid-binding globulin	1.09	6.52e-18	1.92
P01008	SERPINC1	Antithrombin-III	-0.383	3.64e-16	-1.91

UniProt	*Gene*	*Description*	*coef*	p_{adj}	*ES*
P02774;P02774-3	GC	Vitamin D-binding protein	0.388	8.44e-16	1.78
Q9UGM5	FETUB	Fetuin-B	1.56	2e-14	1.81
P04196	HRG	Histidine-rich glycoprotein	-0.646	5.69e-11	-1.66
P00747	PLG	Plasminogen	0.361	7.85e-11	1.61
P02763	ORM1	Alpha-1-acid glycoprotein 1	-0.72	1.75e-10	-1.69
P20742	PZP	Pregnancy zone protein	1.33	5.1e-10	1.58
P51884	LUM	Lumican	-0.597	7.85e-10	-1.53
P05155;P05155-3	SERPING1	Plasma protease C1 inhibitor	-1.49	2.65e-09	-1.62
P01042;P01042-2	KNG1	Kininogen-1	0.323	3.81e-09	1.55
P05543	SERPINA7	Thyroxine-binding globulin	0.76	6.58e-09	1.48
P04217;P04217-2	A1BG	Alpha-1B-glycoprotein	0.406	2.36e-08	1.55
P05452	CLEC3B	Tetranectin	-0.462	1.75e-07	-1.44
P01042-2	KNG1	Isoform LMW of Kininogen-1	0.583	1.76e-07	1.44
P07225	PROS1	Vitamin K-dependent protein S	-0.427	1.85e-07	-1.39
P20851;P20851-2	C4BPB	C4b-binding protein beta chain	-0.674	3.93e-07	-1.39
P04003	C4BPA	C4b-binding protein alpha chain	-0.39	1.28e-06	-1.28
P01009	SERPINA1	Alpha-1-antitrypsin	0.385	3.42e-06	1.24
P04004	VTN	Vitronectin	0.299	4.65e-06	1.17
P13671	C6	Complement component C6	-0.388	5.19e-05	-1.2
P02768	ALB	Serum albumin	-0.241	7.19e-05	-1.19
P19652	ORM2	Alpha-1-acid glycoprotein 2	-0.508	0.000176	-1.17
P05546	SERPIND1	Heparin cofactor 2	0.382	0.000959	1.07
P00738	HP	Haptoglobin	-0.677	0.00203	-1.06
Q16610;Q16610-4	ECM1	Extracellular matrix protein 1	-0.523	0.00272	-1.05
P02747	C1QC	Complement C1q subcomponent subunit C	-0.327	0.00503	-1.02
P01011	SERPINA3	Alpha-1-antichymotrypsin	-0.278	0.00741	-0.996
O14791;O14791-2;O14791-3	APOL1	Apolipoprotein L1	0.428	0.00828	0.929
P02656	APOC3	Apolipoprotein C-III	0.427	0.0104	0.985
P00748	F12	Coagulation factor XII	0.446	0.014	0.951
P07358	C8B	Complement component C8 beta chain	-0.345	0.0163	-0.964
P08571	CD14	Monocyte differentiation antigen CD14	-0.553	0.0324	-0.922
P00736	C1R	Complement C1r subcomponent	-0.268	0.0447	-0.897

Table S19: Proteins significantly associated with ATC level 3 medication *CONTRACEPTIVES FOR TOPICAL USE* (ATC3 G02B). *coef* and p_{adj}: coefficient (representing the differential abundance in log2 scale) and p-value adjusted for multiple hypothesis testing. *ES* effect size. Proteins are ordered by p-value.

UniProt	*Gene*	*Description*	*coef*	p_{adj}	*ES*
P01019	AGT	Angiotensinogen	0.685	4.22e-20	1.04
P00450	CP	Ceruloplasmin	0.272	4.55e-10	0.788
Q96PD5	PGLYRP2	N-acetylmuramoyl-L-alanine amidase	-0.29	1.01e-07	-0.766
P02774;P02774-3	GC	Vitamin D-binding protein	0.164	1.04e-07	0.756
P08185	SERPINA6	Corticosteroid-binding globulin	0.41	8.82e-07	0.722
P00747	PLG	Plasminogen	0.17	1.56e-06	0.759
P80108	GPLD1	Phosphatidylinositol-glycan-specific phospholipase D	0.339	1.94e-06	0.786
P04278	SHBG	Sex hormone-binding globulin	0.799	3.64e-06	0.647
P04003	C4BPA	C4b-binding protein alpha chain	-0.222	5.5e-06	-0.731
P05543	SERPINA7	Thyroxine-binding globulin	0.333	0.000177	0.65
P01876	IGHA1	Immunoglobulin heavy constant alpha 1	-0.446	0.000594	-0.677
P02760	AMBP	Protein AMBP	-0.137	0.00208	-0.631
P01009	SERPINA1	Alpha-1-antitrypsin	0.177	0.00245	0.57
Q9UGM5	FETUB	Fetuin-B	0.479	0.00278	0.555
P05546	SERPIND1	Heparin cofactor 2	0.213	0.00358	0.599
P01008	SERPINC1	Antithrombin-III	-0.104	0.00951	-0.519
P02765	AHSG	Alpha-2-HS-glycoprotein	0.148	0.0233	0.533
P02787	TF	Serotransferrin	0.123	0.0487	0.489
P02753	RBP4	Retinol-binding protein 4	0.162	0.0489	0.48

Table S20: Proteins significantly associated with ATC level 3 medication *ANTIINFLAMMATORY AND ANTIRHEUMATIC PRODUCTS, NON-STEROIDS* (ATC3 M01A). $coef$ and p_{adj}: coefficient (representing the differential abundance in log2 scale) and p-value adjusted for multiple hypothesis testing. *ES* effect size. Proteins are ordered by p-value.

UniProt	*Gene*	*Description*	$coef$	p_{adj}	*ES*
P02748	C9	Complement component C9	0.249	0.00066	0.632
P01011	SERPINA3	Alpha-1-antichymotrypsin	0.161	0.00752	0.579
P02750	LRG1	Leucine-rich alpha-2-glycoprotein	0.236	0.00804	0.542

Table S21: Proteins significantly associated with ATC level 3 medication *DOPAMINERGIC AGENTS* (ATC3 N04B). $coef$ and p_{adj}: coefficient (representing the differential abundance in log2 scale) and p-value adjusted for multiple hypothesis testing. *ES* effect size. Proteins are ordered by p-value.

UniProt	*Gene*	*Description*	$coef$	p_{adj}	*ES*
P02748	C9	Complement component C9	0.364	0.0157	0.921

Table S22: Proteins significantly associated with ATC level 3 medication *BLOOD GLUCOSE LOWERING DRUGS, EXCL. INSULINS* (ATC3 A10B). $coef$ and p_{adj}: coefficient (representing the differential abundance in log2 scale) and p-value adjusted for multiple hypothesis testing. *ES* effect size. Proteins are ordered by p-value.

UniProt	*Gene*	*Description*	$coef$	p_{adj}	*ES*
P06727	APOA4	Apolipoprotein A-IV	0.332	1.44e-06	0.769
P02649	APOE	Apolipoprotein E	-0.3	0.00166	-0.623
P05090	APOD	Apolipoprotein D	-0.224	0.00331	-0.556
P02654	APOC1	Apolipoprotein C-I	-0.428	0.0207	-0.539

Table S23: Proteins significantly associated with ATC level 3 medication *ANTIGOUT PREPARATIONS* (ATC3 M04A). $coef$ and p_{adj}: coefficient (representing the differential abundance in log2 scale) and p-value adjusted for multiple hypothesis testing. *ES* effect size. Proteins are ordered by p-value.

UniProt	*Gene*	*Description*	$coef$	p_{adj}	*ES*
P00734	F2	Prothrombin	-0.137	0.00415	-0.688
P02763	ORM1	Alpha-1-acid glycoprotein 1	0.258	0.032	0.604

Table S24: Proteins significantly associated with ATC level 3 medication *ANTITHROMBOTIC AGENTS* (ATC3 B01A). $coef$ and p_{adj}: coefficient (representing the differential abundance in log2 scale) and p-value adjusted for multiple hypothesis testing. *ES* effect size. Proteins are ordered by p-value.

UniProt	*Gene*	*Description*	$coef$	p_{adj}	*ES*
P00734	F2	Prothrombin	-0.136	6.69e-15	-0.684
P01023	A2M	Alpha-2-macroglobulin	0.224	8.09e-10	0.54

Table S25: Proteins significantly associated with ATC level 3 medication *BETA BLOCKING AGENTS* (ATC3 C07A). $coef$ and p_{adj}: coefficient (representing the differential abundance in log2 scale) and p-value adjusted for multiple hypothesis testing. ES effect size. Proteins are ordered by p-value.

UniProt	*Gene*	*Description*	$coef$	p_{adj}	ES
P00734	F2	Prothrombin	-0.106	3.84e-07	-0.532

Table S26: Proteins significantly associated with ATC level 3 medication *DRUGS USED IN BENIGN PROSTATIC HYPERTROPHY* (ATC3 G04C). $coef$ and p_{adj}: coefficient (representing the differential abundance in log2 scale) and p-value adjusted for multiple hypothesis testing. ES effect size. Proteins are ordered by p-value.

UniProt	*Gene*	*Description*	$coef$	p_{adj}	ES
P04278	SHBG	Sex hormone-binding globulin	0.647	0.00171	0.523
A0A0J9YX35	IGHV3-64D	Immunoglobulin heavy variable 3-64D	0.342	0.00259	0.647
P01023	A2M	Alpha-2-macroglobulin	0.246	0.00371	0.593
P02753	RBP4	Retinol-binding protein 4	-0.173	0.0273	-0.515

Table S27: Proteins significantly associated with ATC level 3 medication *ANTIEPILEPTICS* (ATC3 N03A). $coef$ and p_{adj}: coefficient (representing the differential abundance in log2 scale) and p-value adjusted for multiple hypothesis testing. ES effect size. Proteins are ordered by p-value.

UniProt	*Gene*	*Description*	$coef$	p_{adj}	ES
P80108	GPLD1	Phosphatidylinositol-glycan-specific phospholipase D	0.452	8.27e-09	1.05

Table S28: Proteins significantly associated with ATC level 3 medication *ADRENERGICS, INHALANTS* (ATC3 R03A). $coef$ and p_{adj}: coefficient (representing the differential abundance in log2 scale) and p-value adjusted for multiple hypothesis testing. ES effect size. Proteins are ordered by p-value.

UniProt	*Gene*	*Description*	$coef$	p_{adj}	ES
P00488	F13A1	Coagulation factor XIII A chain	-0.483	0.0186	-0.719

Table S29: Proteins significantly associated with ATC level 3 medication *CALCIUM* (ATC3 A12A). $coef$ and p_{adj}: coefficient (representing the differential abundance in log2 scale) and p-value adjusted for multiple hypothesis testing. ES effect size. Proteins are ordered by p-value.

UniProt	*Gene*	*Description*	$coef$	p_{adj}	ES
P01859	IGHG2	Immunoglobulin heavy constant gamma 2	-0.253	0.00388	-0.536
P06727	APOA4	Apolipoprotein A-IV	0.185	0.0469	0.43

Table S30: Proteins significantly associated with ATC level 3 medication *LIPID MODIFYING AGENTS, PLAIN* (ATC3 C10A). $coef$ and p_{adj}: coefficient (representing the differential abundance in log2 scale) and p-value adjusted for multiple hypothesis testing. ES effect size. Proteins are ordered by p-value.

UniProt	*Gene*	*Description*	$coef$	p_{adj}	ES
P04114	APOB	Apolipoprotein B-100	-0.264	1.36e-12	-0.649

Table S31: Proteins significantly associated with ATC level 3 medication *ANGIOTENSIN II ANTAGONISTS, COMBINATIONS* (ATC3 C09D). $coef$ and p_{adj}: coefficient (representing the differential abundance in log2 scale) and p-value adjusted for multiple hypothesis testing. ES effect size. Proteins are ordered by p-value.

UniProt	*Gene*	*Description*	$coef$	p_{adj}	ES
P01024	C3	Complement C3	0.0692	0.0322	0.346

Table S32: Proteins significantly associated with ATC level 3 medication *ANGIOTENSIN II ANTAGONISTS, PLAIN* (ATC3 C09C). $coef$ and p_{adj}: coefficient (representing the differential abundance in log2 scale) and p-value adjusted for multiple hypothesis testing. ES effect size. Proteins are ordered by p-value.

UniProt	*Gene*	*Description*	$coef$	p_{adj}	ES
P01602	IGKV1-5	Immunoglobulin kappa variable 1-5	0.237	0.0284	0.441

ATC level 4 medications

Associations were evaluated for 34 ATC level 4 medications taken on a regular basis (at least twice per week) by more than 14 study participants. The considered medications, numbers of participants and significant proteins are shown in the table below.

Table S33: Overview of association results for ATC level 4 medications in the CHRIS study subset. Only medications taken on a regular basis by more than 14 of the in total 3,632 study participants were considered. Columns *Participants* and *Proteins* list the number of participants taking the medication and number of significantly associated proteins.

ATC	Name	Participants	Proteins
H03AA	Thyroid hormones	262	0
G03AA, G03FA	Progestogens and estrogens, fixed combinations	260	52
B01AC	Platelet aggregation inhibitors excl. heparin	211	1
C10AA	HMG CoA reductase inhibitors	193	1
C09AA	ACE inhibitors, plain	156	0
C07AB	Beta blocking agents, selective	135	0
C09DA	Angiotensin II antagonists and diuretics	109	0
N06AB	Selective serotonin reuptake inhibitors	94	0
A02BC	Proton pump inhibitors	92	0
C08CA	Dihydropyridine derivatives	91	0
C09CA	Angiotensin II antagonists, plain	74	1
A12AX	Calcium, combinations with vitamin D and/or other drugs	62	1
C09BA	ACE inhibitors and diuretics	59	0
N05BA, N05CD	Benzodiazepine derivatives	51	0

ATC	Name	Participants	Proteins
G04CA, C02CA	Alpha-adrenoreceptor antagonists	48	5
A10BA	Biguanides	43	4
N06AX	Other antidepressants	41	0
B01AA	Vitamin K antagonists	37	14
M04AA	Preparations inhibiting uric acid production	36	0
G03AB, G03FB	Progestogens and estrogens, sequential preparations	34	36
R03AK	Adrenergics in combination with corticosteroids or other drugs, excl. anticholinergics	27	1
G02BA	Intrauterine contraceptives	25	0
M01AE	Propionic acid derivatives	24	0
S01ED	Beta blocking agents	21	0
G02BB	Intravaginal contraceptives	21	35
N03AX	Other antiepileptics	21	0
H02AB, R03BA	Glucocorticoids	18	2
M01AB	Acetic acid derivatives and related substances	16	0
G03HB	Antiandrogens and estrogens	16	38

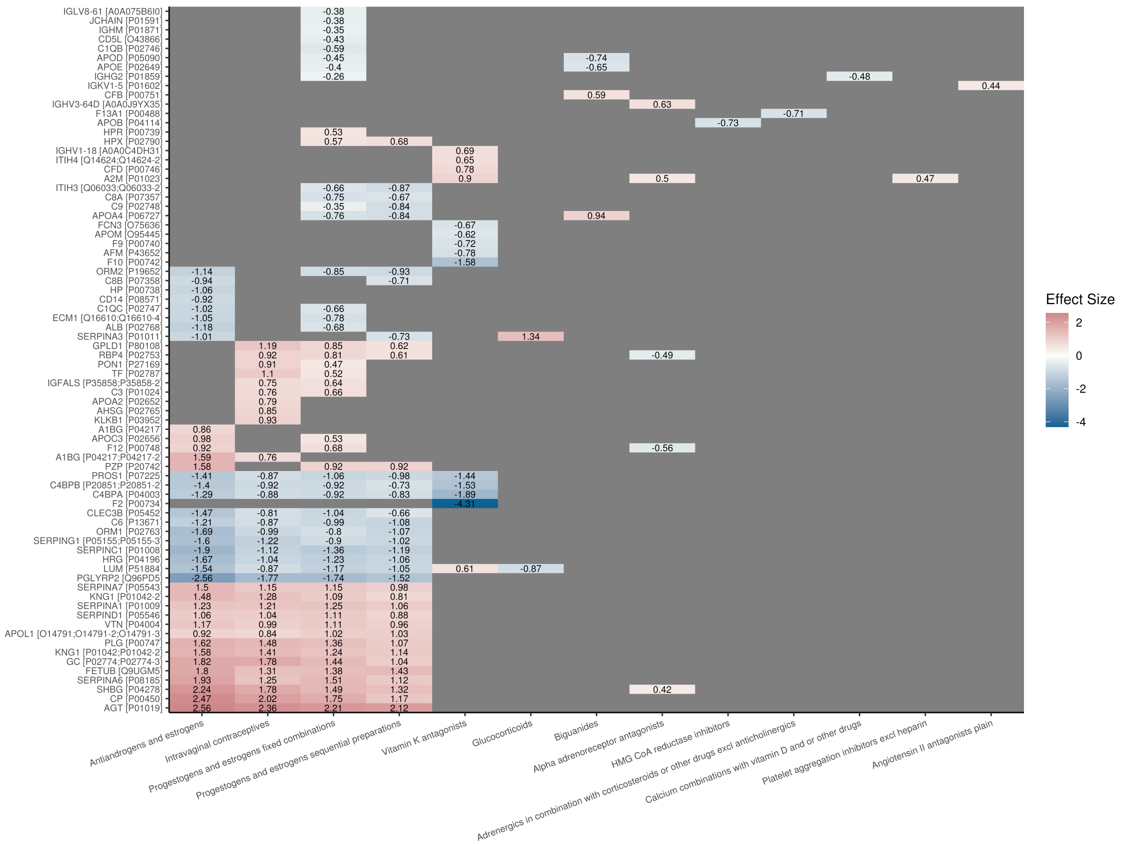

Figure S8: Significant associations between proteins (rows) and ATC level 4 medications (columns). Effect sizes (as a number and color coded) are only shown for significant associations. Ordering of rows and columns represent the result from a hierarchical cluster analysis (Ward clustering of an Euclidean dissimilarity matrix).

Table S34: Proteins significantly associated with ATC level 4 medication *Progestogens and estrogens, fixed combinations* (ATC4 G03AA, G03FA). $coef$ and p_{adj}: coefficient (representing the differential abundance in log2 scale) and p-value adjusted for multiple hypothesis testing. ES effect size. Proteins are ordered by p-value.

UniProt	*Gene*	*Description*	$coef$	p_{adj}	ES
P01019	AGT	Angiotensinogen	1.46	0	2.21
P00450	CP	Ceruloplasmin	0.604	5.58e-212	1.75
Q96PD5	PGLYRP2	N-acetylmuramoyl-L-alanine amidase	-0.658	4.86e-174	-1.74
P04278	SHBG	Sex hormone-binding globulin	1.84	4.15e-149	1.49
P08185	SERPINA6	Corticosteroid-binding globulin	0.86	6.93e-135	1.51
P02774;P02774-3	GC	Vitamin D-binding protein	0.314	6.19e-127	1.44
Q9UGM5	FETUB	Fetuin-B	1.2	1.16e-104	1.38
P01008	SERPINC1	Antithrombin-III	-0.273	4.67e-101	-1.36
P00747	PLG	Plasminogen	0.304	7.55e-97	1.36
P01009	SERPINA1	Alpha-1-antitrypsin	0.386	2.42e-82	1.25
P04196	HRG	Histidine-rich glycoprotein	-0.479	4.06e-76	-1.23
P01042;P01042-2	KNG1	Kininogen-1	0.257	3.53e-75	1.24
P51884	LUM	Lumican	-0.456	3.69e-74	-1.17
P04004	VTN	Vitronectin	0.285	1.71e-73	1.11
P05543	SERPINA7	Thyroxine-binding globulin	0.592	4.32e-70	1.15
P07225	PROS1	Vitamin K-dependent protein S	-0.326	1.93e-58	-1.06
P05546	SERPIND1	Heparin cofactor 2	0.395	5.03e-58	1.11
P01042-2	KNG1	Isoform LMW of Kininogen-1	0.441	1.25e-56	1.09
O14791;O14791-2;O14791-3	APOL1	Apolipoprotein L1	0.471	1.34e-52	1.02
P05452	CLEC3B	Tetranectin	-0.333	7.97e-52	-1.04
P13671	C6	Complement component C6	-0.321	1.11e-47	-0.993
P04003	C4BPA	C4b-binding protein alpha chain	-0.279	7.19e-47	-0.918
P20742	PZP	Pregnancy zone protein	0.771	5.44e-44	0.917
P20851;P20851-2	C4BPB	C4b-binding protein beta chain	-0.445	1.64e-42	-0.92
P05155;P05155-3	SERPING1	Plasma protease C1 inhibitor	-0.834	5.03e-38	-0.901
P80108	GPLD1	Phosphatidylinositol-glycan-specific phospholipase D	0.367	7.78e-36	0.851
P02753	RBP4	Retinol-binding protein 4	0.274	3.2e-35	0.813
P19652	ORM2	Alpha-1-acid glycoprotein 2	-0.369	1.77e-33	-0.849
P02763	ORM1	Alpha-1-acid glycoprotein 1	-0.342	4.68e-31	-0.801
P06727	APOA4	Apolipoprotein A-IV	-0.328	4.31e-28	-0.76
Q16610;Q16610-4	ECM1	Extracellular matrix protein 1	-0.386	7.84e-27	-0.777
P07357	C8A	Complement component C8 alpha chain	-0.213	1.19e-24	-0.751
P35858;P35858-2	IGFALS	Insulin-like growth factor-binding protein complex acid labile subunit	0.284	6.38e-24	0.641
P01024	C3	Complement C3	0.132	4.03e-23	0.661
Q06033;Q06033-2	ITIH3	Inter-alpha-trypsin inhibitor heavy chain H3	-0.38	4.24e-22	-0.662
P02768	ALB	Serum albumin	-0.137	5.28e-22	-0.68
P00748	F12	Coagulation factor XII	0.317	1.44e-20	0.676
P02747	C1QC	Complement C1q subcomponent subunit C	-0.212	1.7e-19	-0.665
P02746	C1QB	Complement C1q subcomponent subunit B	-0.196	3.81e-15	-0.594
P02790	HPX	Hemopexin	0.115	1.08e-14	0.568
P02787	TF	Serotransferrin	0.13	1.57e-13	0.516
P00739	HPR	Haptoglobin-related protein	0.32	5.35e-12	0.529
P02656	APOC3	Apolipoprotein C-III	0.229	5.58e-12	0.527
P05090	APOD	Apolipoprotein D	-0.18	6.41e-10	-0.445
P27169	PON1	Serum paraoxonase/arylesterase 1	0.204	1.31e-09	0.467
O43866	CD5L	CD5 antigen-like	-0.32	4.16e-08	-0.427
P02649	APOE	Apolipoprotein E	-0.194	1.25e-06	-0.403
A0A075B6I0	IGLV8-61	Immunoglobulin lambda variable 8-61	-0.299	8.51e-06	-0.381
P01591	JCHAIN	Immunoglobulin J chain	-0.233	8.91e-06	-0.376
P02748	C9	Complement component C9	-0.139	1.72e-05	-0.353

UniProt	*Gene*	*Description*	*coef*	p_{adj}	*ES*
P01871	IGHM	Immunoglobulin heavy constant mu	-0.264	3.54e-05	-0.347
P01859	IGHG2	Immunoglobulin heavy constant gamma 2	-0.124	0.0273	-0.263

Table S35: Proteins significantly associated with ATC level 4 medication *Antiandrogens and estrogens* (ATC4 G03HB). *coef* and p_{adj}: coefficient (representing the differential abundance in log2 scale) and p-value adjusted for multiple hypothesis testing. *ES* effect size. Proteins are ordered by p-value.

UniProt	*Gene*	*Description*	*coef*	p_{adj}	*ES*
P01019	AGT	Angiotensinogen	1.69	7.78e-46	2.56
P00450	CP	Ceruloplasmin	0.855	1.26e-37	2.47
Q96PD5	PGLYRP2	N-acetylmuramoyl-L-alanine amidase	-0.968	1.31e-32	-2.56
P04278	SHBG	Sex hormone-binding globulin	2.76	1.47e-28	2.24
P08185	SERPINA6	Corticosteroid-binding globulin	1.1	1.91e-18	1.93
P02774;P02774-3	GC	Vitamin D-binding protein	0.396	6.8e-17	1.82
P01008	SERPINC1	Antithrombin-III	-0.38	4.52e-16	-1.9
Q9UGM5	FETUB	Fetuin-B	1.56	1.66e-14	1.8
P00747	PLG	Plasminogen	0.364	4.01e-11	1.62
P04196	HRG	Histidine-rich glycoprotein	-0.647	4.9e-11	-1.67
P02763	ORM1	Alpha-1-acid glycoprotein 1	-0.72	1.18e-10	-1.69
P51884	LUM	Lumican	-0.602	3.31e-10	-1.54
P20742	PZP	Pregnancy zone protein	1.33	3.94e-10	1.58
P01042;P01042-2	KNG1	Kininogen-1	0.329	1.11e-09	1.58
P05543	SERPINA7	Thyroxine-binding globulin	0.769	2.9e-09	1.5
P05155;P05155-3	SERPING1	Plasma protease C1 inhibitor	-1.48	2.96e-09	-1.6
P04217;P04217-2	A1BG	Alpha-1B-glycoprotein	0.417	5.9e-09	1.59
P01042-2	KNG1	Isoform LMW of Kininogen-1	0.599	4.97e-08	1.48
P07225	PROS1	Vitamin K-dependent protein S	-0.433	6.2e-08	-1.41
P05452	CLEC3B	Tetranectin	-0.47	7.16e-08	-1.47
P20851;P20851-2	C4BPB	C4b-binding protein beta chain	-0.676	1.98e-07	-1.4
P04003	C4BPA	C4b-binding protein alpha chain	-0.393	4.83e-07	-1.29
P04004	VTN	Vitronectin	0.3	3.32e-06	1.17
P01009	SERPINA1	Alpha-1-antitrypsin	0.382	3.81e-06	1.23
P13671	C6	Complement component C6	-0.39	3.91e-05	-1.21
P02768	ALB	Serum albumin	-0.237	1e-04	-1.18
P19652	ORM2	Alpha-1-acid glycoprotein 2	-0.495	0.000299	-1.14
P05546	SERPIND1	Heparin cofactor 2	0.375	0.00127	1.06
P00738	HP	Haptoglobin	-0.675	0.00198	-1.06
Q16610;Q16610-4	ECM1	Extracellular matrix protein 1	-0.521	0.00276	-1.05
P01011	SERPINA3	Alpha-1-antichymotrypsin	-0.282	0.00505	-1.01
P02747	C1QC	Complement C1q subcomponent subunit C	-0.325	0.00519	-1.02
O14791;O14791-2;O14791-3	APOL1	Apolipoprotein L1	0.425	0.00856	0.922
P02656	APOC3	Apolipoprotein C-III	0.427	0.00995	0.984
P00748	F12	Coagulation factor XII	0.434	0.021	0.925
P07358	C8B	Complement component C8 beta chain	-0.337	0.0216	-0.943
P08571	CD14	Monocyte differentiation antigen CD14	-0.552	0.0311	-0.921
P04217	A1BG	Alpha-1B-glycoprotein	0.197	0.0475	0.861

Table S36: Proteins significantly associated with ATC level 4 medication *Progestogens and estrogens, sequential preparations* (ATC4 G03AB, G03FB). *coef* and p_{adj}: coefficient (representing the differential abundance in log2 scale) and p-value adjusted for multiple hypothesis testing. *ES* effect size. Proteins are ordered by p-value.

UniProt	*Gene*	*Description*	*coef*	p_{adj}	*ES*
P01019	AGT	Angiotensinogen	1.4	8.3e-65	2.12
Q96PD5	PGLYRP2	N-acetylmuramoyl-L-alanine amidase	-0.578	3.1e-24	-1.52
P04278	SHBG	Sex hormone-binding globulin	1.63	8.28e-21	1.32

UniProt	*Gene*	*Description*	*coef*	p_{adj}	*ES*
Q9UGM5	FETUB	Fetuin-B	1.23	3.74e-19	1.43
P00450	CP	Ceruloplasmin	0.405	1.29e-17	1.17
P01008	SERPINC1	Antithrombin-III	-0.239	3.58e-13	-1.19
P08185	SERPINA6	Corticosteroid-binding globulin	0.638	7.15e-13	1.12
P02774;P02774-3	GC	Vitamin D-binding protein	0.226	2.54e-11	1.04
P01042;P01042-2	KNG1	Kininogen-1	0.236	1.78e-10	1.14
P00747	PLG	Plasminogen	0.241	4.11e-10	1.07
P01009	SERPINA1	Alpha-1-antitrypsin	0.327	8.83e-10	1.06
P51884	LUM	Lumican	-0.409	8.99e-10	-1.05
P04196	HRG	Histidine-rich glycoprotein	-0.412	3.13e-09	-1.06
P13671	C6	Complement component C6	-0.35	3.93e-09	-1.08
P04004	VTN	Vitronectin	0.247	4.56e-09	0.965
P02763	ORM1	Alpha-1-acid glycoprotein 1	-0.455	8.83e-09	-1.07
O14791;O14791-2;O14791-3	APOL1	Apolipoprotein L1	0.475	1.3e-08	1.03
P05543	SERPINA7	Thyroxine-binding globulin	0.504	3.11e-08	0.983
P07225	PROS1	Vitamin K-dependent protein S	-0.301	5.71e-08	-0.978
P05155;P05155-3	SERPING1	Plasma protease C1 inhibitor	-0.942	1.12e-07	-1.02
P20742	PZP	Pregnancy zone protein	0.771	6.29e-07	0.918
P19652	ORM2	Alpha-1-acid glycoprotein 2	-0.404	3.34e-06	-0.928
P04003	C4BPA	C4b-binding protein alpha chain	-0.253	5.77e-06	-0.832
Q06033;Q06033-2	ITIH3	Inter-alpha-trypsin inhibitor heavy chain H3	-0.5	6.69e-06	-0.872
P05546	SERPIND1	Heparin cofactor 2	0.313	1.22e-05	0.881
P06727	APOA4	Apolipoprotein A-IV	-0.362	3.39e-05	-0.84
P02748	C9	Complement component C9	-0.33	5.02e-05	-0.835
P01042-2	KNG1	Isoform LMW of Kininogen-1	0.326	0.000117	0.806
P20851;P20851-2	C4BPB	C4b-binding protein beta chain	-0.352	0.000722	-0.728
P01011	SERPINA3	Alpha-1-antichymotrypsin	-0.205	0.00201	-0.734
P07358	C8B	Complement component C8 beta chain	-0.253	0.00562	-0.708
P02790	HPX	Hemopexin	0.138	0.00628	0.683
P05452	CLEC3B	Tetranectin	-0.212	0.00702	-0.663
P02753	RBP4	Retinol-binding protein 4	0.205	0.012	0.61
P07357	C8A	Complement component C8 alpha chain	-0.189	0.0138	-0.669
P80108	GPLD1	Phosphatidylinositol-glycan-specific phospholipase D	0.269	0.0154	0.623

Table S37: Proteins significantly associated with ATC level 4 medication *Intravaginal contraceptives* (ATC4 G02BB). *coef* and p_{adj}: coefficient (representing the differential abundance in log2 scale) and p-value adjusted for multiple hypothesis testing. *ES* effect size. Proteins are ordered by p-value.

UniProt	*Gene*	*Description*	*coef*	p_{adj}	*ES*
P01019	AGT	Angiotensinogen	1.56	1.9e-50	2.36
P00450	CP	Ceruloplasmin	0.698	1.22e-32	2.02
P04278	SHBG	Sex hormone-binding globulin	2.2	1.61e-23	1.78
P02774;P02774-3	GC	Vitamin D-binding protein	0.388	4.43e-21	1.78
Q96PD5	PGLYRP2	N-acetylmuramoyl-L-alanine amidase	-0.67	3.19e-20	-1.77
P00747	PLG	Plasminogen	0.331	4.95e-12	1.48
P01042;P01042-2	KNG1	Kininogen-1	0.293	4.85e-10	1.41
Q9UGM5	FETUB	Fetuin-B	1.13	7.51e-10	1.31
P08185	SERPINA6	Corticosteroid-binding globulin	0.711	7.69e-10	1.25
P01009	SERPINA1	Alpha-1-antitrypsin	0.376	6.31e-08	1.21
P01042-2	KNG1	Isoform LMW of Kininogen-1	0.519	8.48e-08	1.28
P01008	SERPINC1	Antithrombin-III	-0.225	6.09e-07	-1.12
P05543	SERPINA7	Thyroxine-binding globulin	0.591	6.14e-07	1.15
P05155;P05155-3	SERPING1	Plasma protease C1 inhibitor	-1.13	8.33e-07	-1.22
P80108	GPLD1	Phosphatidylinositol-glycan-specific phospholipase D	0.512	8.72e-07	1.19
P02787	TF	Serotransferrin	0.278	5.2e-06	1.1
P04004	VTN	Vitronectin	0.254	1.09e-05	0.989
P04196	HRG	Histidine-rich glycoprotein	-0.404	3.21e-05	-1.04
P05546	SERPIND1	Heparin cofactor 2	0.37	8.61e-05	1.04
P02763	ORM1	Alpha-1-acid glycoprotein 1	-0.425	0.000207	-0.995

UniProt	*Gene*	*Description*	*coef*	p_{adj}	*ES*
P02753	RBP4	Retinol-binding protein 4	0.309	0.000439	0.917
P04003	C4BPA	C4b-binding protein alpha chain	-0.266	0.000717	-0.876
P20851;P20851-2	C4BPB	C4b-binding protein beta chain	-0.444	0.000796	-0.918
P51884	LUM	Lumican	-0.337	0.00104	-0.866
P07225	PROS1	Vitamin K-dependent protein S	-0.267	0.00165	-0.868
P03952	KLKB1	Plasma kallikrein	0.297	0.00219	0.93
P27169	PON1	Serum paraoxonase/arylesterase 1	0.395	0.00309	0.906
P13671	C6	Complement component C6	-0.281	0.00346	-0.869
O14791;O14791-2;O14791-3	APOL1	Apolipoprotein L1	0.386	0.00453	0.838
P02765	AHSG	Alpha-2-HS-glycoprotein	0.237	0.0058	0.851
P35858;P35858-2	IGFALS	Insulin-like growth factor-binding protein complex acid labile subunit	0.33	0.0104	0.746
P05452	CLEC3B	Tetranectin	-0.259	0.0132	-0.81
P01024	C3	Complement C3	0.151	0.0177	0.758
P02652	APOA2	Apolipoprotein A-II	0.172	0.0312	0.786
P04217;P04217-2	A1BG	Alpha-1B-glycoprotein	0.199	0.0456	0.759

Table S38: Proteins significantly associated with ATC level 4 medication *Vitamin K antagonists* (ATC4 B01AA). *coef* and p_{adj}: coefficient (representing the differential abundance in log2 scale) and p-value adjusted for multiple hypothesis testing. *ES* effect size. Proteins are ordered by p-value.

UniProt	*Gene*	*Description*	*coef*	p_{adj}	*ES*
P00734	F2	Prothrombin	-0.857	2.35e-164	-4.31
P04003	C4BPA	C4b-binding protein alpha chain	-0.573	3.01e-33	-1.89
P20851;P20851-2	C4BPB	C4b-binding protein beta chain	-0.74	1.12e-19	-1.53
P00742	F10	Coagulation factor X	-0.504	1.28e-19	-1.58
P07225	PROS1	Vitamin K-dependent protein S	-0.442	4e-18	-1.44
P01023	A2M	Alpha-2-macroglobulin	0.373	1.91e-06	0.897
P43652	AFM	Afamin	-0.239	0.000378	-0.776
P00746	CFD	Complement factor D	0.439	0.000418	0.781
O75636	FCN3	Ficolin-3	-0.4	0.00366	-0.669
P00740	F9	Coagulation factor IX	-0.584	0.00379	-0.724
P51884	LUM	Lumican	0.238	0.00796	0.61
A0A0C4DH31	IGHV1-18	Immunoglobulin heavy variable 1-18	0.555	0.00957	0.688
Q14624;Q14624-2	ITIH4	Inter-alpha-trypsin inhibitor heavy chain H4	0.141	0.0158	0.652
O95445	APOM	Apolipoprotein M	-0.217	0.0289	-0.616

Table S39: Proteins significantly associated with ATC level 4 medication *Alpha-adrenoreceptor antagonists* (ATC4 G04CA, C02CA). *coef* and p_{adj}: coefficient (representing the differential abundance in log2 scale) and p-value adjusted for multiple hypothesis testing. *ES* effect size. Proteins are ordered by p-value.

UniProt	*Gene*	*Description*	*coef*	p_{adj}	*ES*
A0A0J9YX35	IGHV3-64D	Immunoglobulin heavy variable 3-64D	0.335	0.00302	0.634
P00748	F12	Coagulation factor XII	-0.264	0.0203	-0.562
P02753	RBP4	Retinol-binding protein 4	-0.166	0.0416	-0.493
P04278	SHBG	Sex hormone-binding globulin	0.524	0.042	0.424
P01023	A2M	Alpha-2-macroglobulin	0.209	0.0433	0.503

Table S40: Proteins significantly associated with ATC level 4 medication *Biguanides* (ATC4 A10BA). *coef* and p_{adj}: coefficient (representing the differential abundance in log2 scale) and p-value adjusted for multiple hypothesis testing. *ES* effect size. Proteins are ordered by p-value.

UniProt	*Gene*	*Description*	*coef*	p_{adj}	*ES*
P06727	APOA4	Apolipoprotein A-IV	0.404	1.77e-07	0.938
P05090	APOD	Apolipoprotein D	-0.299	0.000112	-0.741
P02649	APOE	Apolipoprotein E	-0.314	0.00918	-0.653
P00751	CFB	Complement factor B	0.141	0.0349	0.586

Table S41: Proteins significantly associated with ATC level 4 medication *Glucocorticoids* (ATC4 H02AB, R03BA). *coef* and p_{adj}: coefficient (representing the differential abundance in log2 scale) and p-value adjusted for multiple hypothesis testing. *ES* effect size. Proteins are ordered by p-value.

UniProt	*Gene*	*Description*	*coef*	p_{adj}	*ES*
P01011	SERPINA3	Alpha-1-antichymotrypsin	0.374	1.67e-06	1.34
P51884	LUM	Lumican	-0.341	0.00509	-0.875

Table S42: Proteins significantly associated with ATC level 4 medication *Platelet aggregation inhibitors excl. heparin* (ATC4 B01AC). *coef* and p_{adj}: coefficient (representing the differential abundance in log2 scale) and p-value adjusted for multiple hypothesis testing. *ES* effect size. Proteins are ordered by p-value.

UniProt	*Gene*	*Description*	*coef*	p_{adj}	*ES*
P01023	A2M	Alpha-2-macroglobulin	0.194	1.69e-06	0.467

Table S43: Proteins significantly associated with ATC level 4 medication *HMG CoA reductase inhibitors* (ATC4 C10AA). *coef* and p_{adj}: coefficient (representing the differential abundance in log2 scale) and p-value adjusted for multiple hypothesis testing. *ES* effect size. Proteins are ordered by p-value.

UniProt	*Gene*	*Description*	*coef*	p_{adj}	*ES*
P04114	APOB	Apolipoprotein B-100	-0.295	4.97e-15	-0.726

Table S44: Proteins significantly associated with ATC level 4 medication *Angiotensin II antagonists, plain* (ATC4 C09CA). *coef* and p_{adj}: coefficient (representing the differential abundance in log2 scale) and p-value adjusted for multiple hypothesis testing. *ES* effect size. Proteins are ordered by p-value.

UniProt	*Gene*	*Description*	*coef*	p_{adj}	*ES*
P01602	IGKV1-5	Immunoglobulin kappa variable 1-5	0.237	0.028	0.441

Table S45: Proteins significantly associated with ATC level 4 medication *Calcium, combinations with vitamin D and/or other drugs* (ATC4 A12AX). *coef* and p_{adj}: coefficient (representing the differential abundance in log2 scale) and p-value adjusted for multiple hypothesis testing. *ES* effect size. Proteins are ordered by p-value.

UniProt	*Gene*	*Description*	*coef*	p_{adj}	*ES*
P01859	IGHG2	Immunoglobulin heavy constant gamma 2	-0.224	0.0443	-0.476

Table S46: Proteins significantly associated with ATC level 4 medication *Adrenergics in combination with corticosteroids or other drugs, excl. anticholinergics* (ATC4 R03AK). *coef* and p_{adj}: coefficient (representing the differential abundance in log2 scale) and p-value adjusted for multiple hypothesis testing. *ES* effect size. Proteins are ordered by p-value.

UniProt	*Gene*	*Description*	*coef*	p_{adj}	*ES*
P00488	F13A1	Coagulation factor XIII A chain	-0.474	0.0412	-0.707

Oral Hormonal Contraceptives Shape the Plasma Proteome in Female Study Participants

A comparison of the results from the analysis on the full data set and on the subset of female participants below 40 years of age is shown below. Coefficients for associations with hormonal contraceptive use are highly similar between the two analyses and p-values highly related (same rank, but difference in the values due to the differences in statistical power from the two analyses).

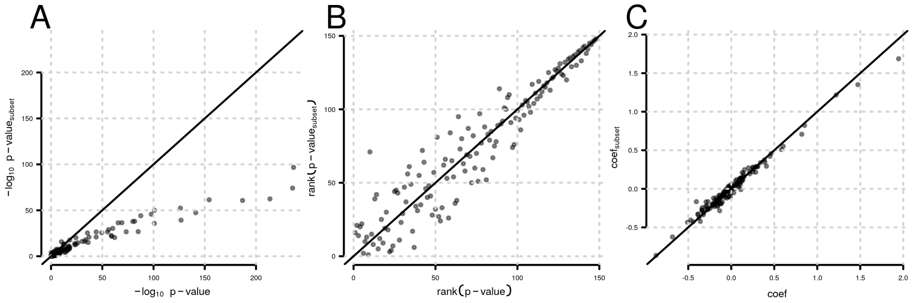

Figure S9: Comparison of results for association with hormonal contraceptive use from the analysis of the full data set (x-axis) and the analysis on the subset of female participants below 40 years of age (y axis). Shown are -log10 of p-values (A), ranks of p-values (B) and coefficients (C).

Hormonal Contraceptive Use Induces Similar Proteomics Changes in an Independent Cohort

Influence of hormonal contraceptives on the serum proteome of female study participants below the age of 40 was validated using data from an independent cohort, the BASE-II study.

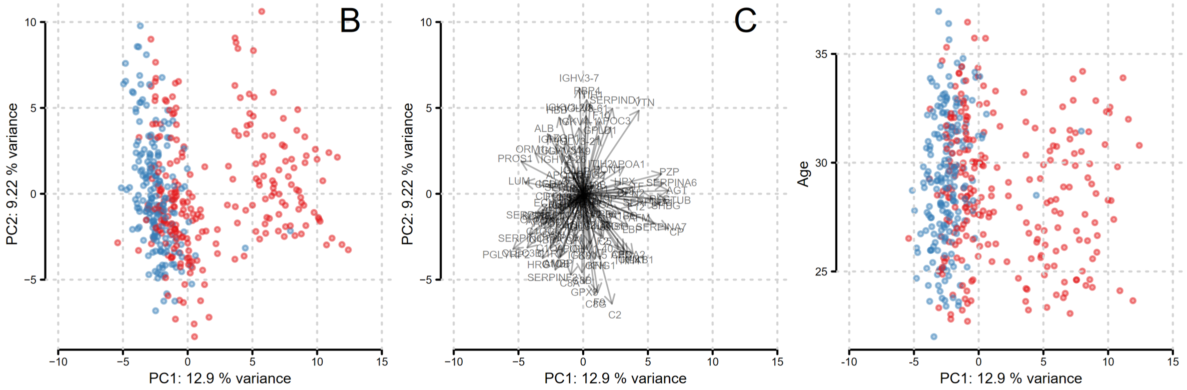

Figure S10: Principal Component Analysis of the serum proteome data from the BASE-II cohort for study participants of age < 40. BASE-II serum proteome data was restricted to the 131 proteins common with CHRIS. Blue and red points indicate male and female participants. Left: grouping of individuals based on PC1 and PC2. Middle: PCA loadings on PC1 and PC2. Each arrow represents a protein with its length and direction indicating the protein's contribution ot the respective principal component. Right: relationship between PC1 (x-axis) and participant's age (y-axis).

Combined Hormonal Contraceptives Containing Ethinylestradiol Have a Stronger Effect Than Those with Natural Estrogens

Table S47: Combined Oral Contraceptive (COC) classes. EE: preparations containing ethinylestradiol, BE: preparations with bioidentical estrogen, P4: progestogen preparations.

ATC5	ATC4.name	COC	count	Preparation.name
G03AA07	levonorgestrel and EE	EE	31	LOETTE, MICROGYNON, LESTRONETTE, EGOGYN, MIRANOVA, NAOMI
G03AA09	desogestrel and EE	EE	10	MERCILON, PRACTIL, PLANUM, DESOREEN
G03AA10	gestodene and EE	EE	104	MINULET, GINODEN, ARIANNA, ESTINETTE, FEDRA, HARMONET, KIPLING, MINESSE, GESTODIOL, GESTODELLE, MELIANE
G03AA12	drospirenone and EE	EE	67	YASMINELLE, LUCINELLE, YASMIN, LUTIZ, LUSINE, YAZ, JASMINELLE, RUBIRA, LUSINELLE
G03AA15	chlormadinone and EE	EE	2	BELARA
G03AA16	dienogest and EE	EE	5	VALETTE, SIBILLA
G03AB05	desogestrel and EE	EE	7	LUCILLE, GRACIAL
G03AB06	gestodene and EE	EE	7	MILVANE, TRIMINULET
G03HB01	cyproterone and estrogen	EE	15	DIANE, VISOFID
G03AC09	desogestrel	P4	6	NACREZ, CERAZETTE
G03AA14	nomegestrol and estradiol	BE	7	ZOELY
G03AB08	dienogest and estradiol	BE	10	KLAIRA, QLAIRA

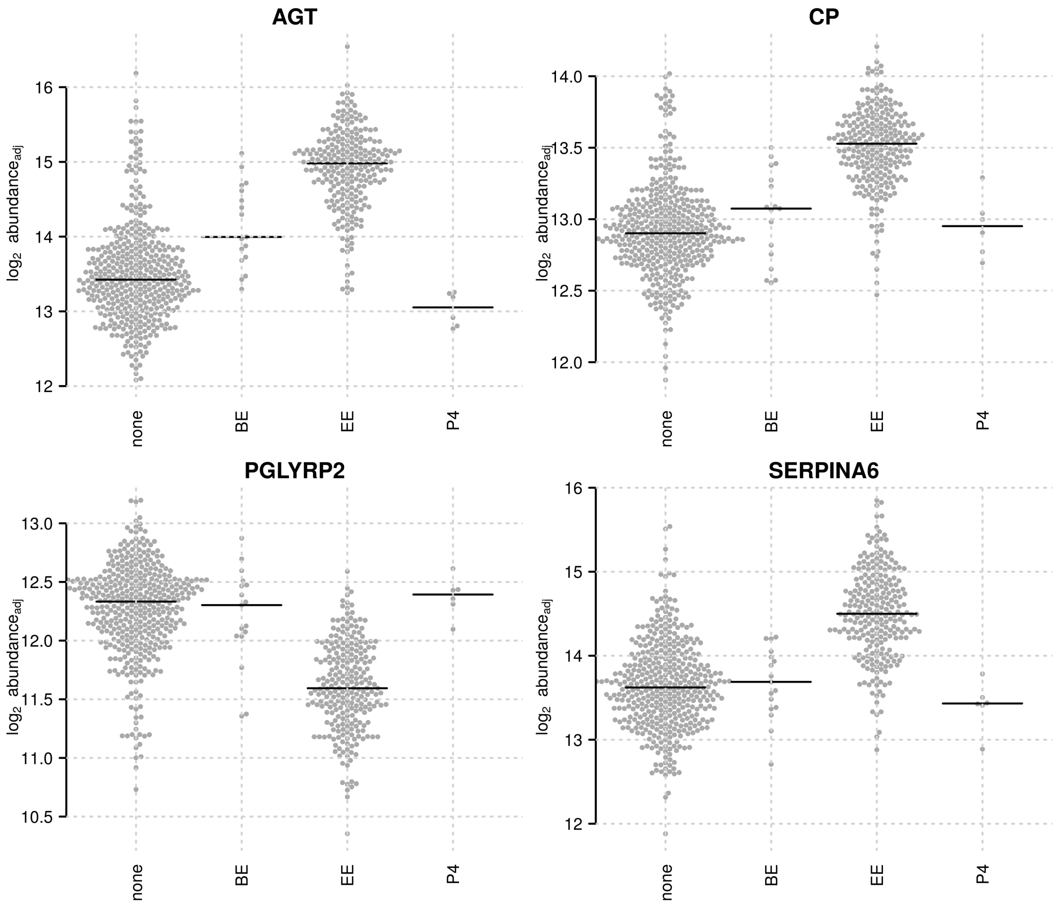

Figure S11: Abundances of proteins significantly associated with hormonal contraceptive use for different types of combined oral contraceptives in the CHRIS study. *EE*: preparations containing ethinylestradiol, *BE*: preparations with bioidentical estrogen, *P4*: progestogen preparations. Shown are individual data points in grey and median abundances per group as black horizontal lines. Abundances are adjusted for age, (categorical) BMI and fasting status.

No Long-Lasting Effects of Hormonal Contraceptives on the Plasma Proteome Observed

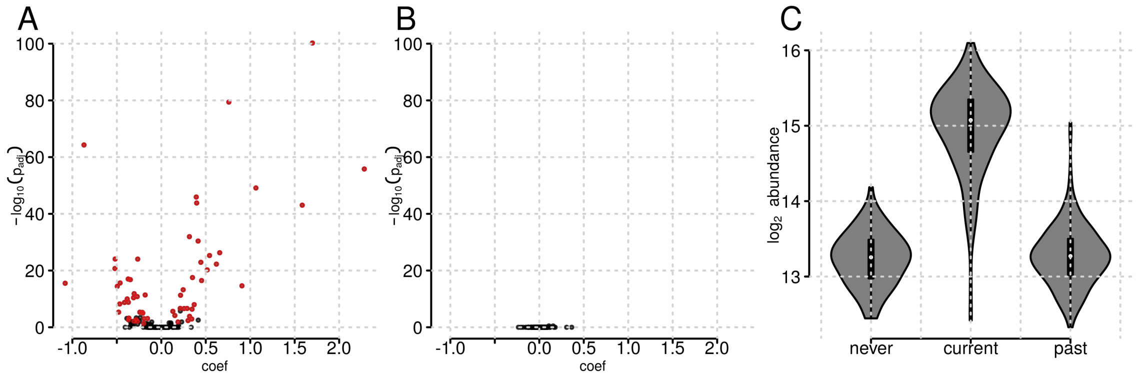

Figure S12: Impact of previous use of hormonal contraceptives on the plasma proteome in the CHRIS study. A: results from the comparison between participants currently taking hormonal contraceptives and those that never took hormonal contraceptives. B: results from the comparison between participants that used hormonal contraceptives in the past and those that never took them. Proteins with significant differences in abundances are highlighted in red. C: abundance of angiotensinogen (AGT) in the three hormonal contraceptive use (HCU) groups.

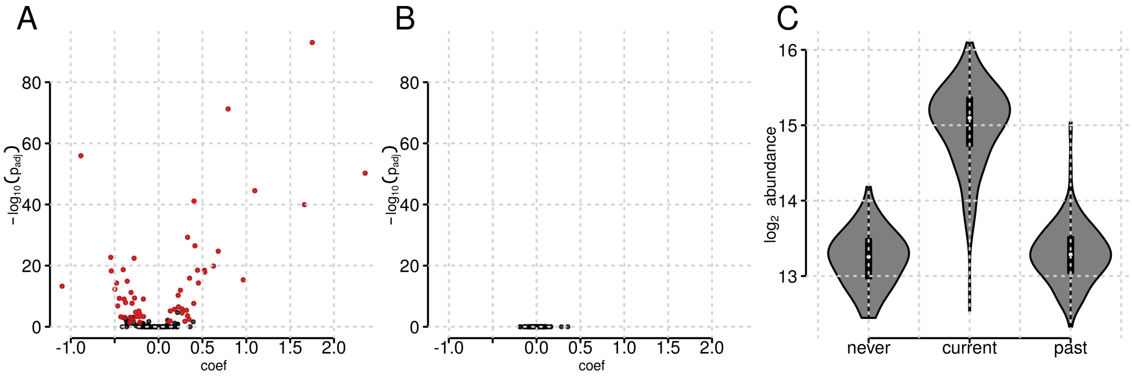

Figure S13: Impact of previous use of hormonal contraceptives on the plasma proteome in the CHRIS study, subset of women who declared to have never been pregnant. A: results from the comparison between participants currently taking hormonal contraceptives and those that never took hormonal contraceptives. B: results from the comparison between participants that used hormonal contraceptives in the past and those that never took them. Proteins with significant differences in abundances are highlighted in red. C: abundance of angiotensinogen (AGT) in the three hormonal contraceptive use (HCU) groups.
