# Supplementary material for: Extensive modulation of the circulating blood proteome by hormonal contraceptive use across two population studies
Source: Commun Med (Lond). 2025 Apr 22;5:131. doi: 10.1038/s43856-025-00856-0 (PMC12015301; doi:10.1038/s43856-025-00856-0)
Supplement: Supplementary file 3 — Description of Additional Supplementary Files [file 43856_2025_856_MOESM3_ESM.pdf]

Description of Additional Supplementary Files

File name: Supplementary Data 1

Description: Spreadsheet (xlsx format) with the results from the association analyses for the CHRIS cohort.

File name: Supplementary Data 2

Description: Spreadsheet (xlsx format) with functional annotations of detected proteins.

File name: Supplementary Data 3

Description: spreadsheet (xlsx format) with the proteins significantly associated with hormonal contraceptive use in women below the age of 40.

File name: Supplementary Data 4

Description: spreadsheet (xlsx format) with results from the association analysis of BASE-II cohort data.

File name: Supplementary Data 5

Description: spreadsheet (xlsx format) with the results for association between proteins and different combined oral contraceptives.

File name: Supplementary Data 6

Description: spreadsheet (xlsx format) with the comparison of results with literature.

File name: Supplementary Data 7

Description: zip archive containing intensity distribution plots for all proteins.
