# Supplementary figures and images for: Extensive modulation of the circulating blood proteome by hormonal contraceptive use across two population studies
Source: Commun Med (Lond). 2025 Apr 22;5:131. doi: 10.1038/s43856-025-00856-0 (PMC12015301; doi:10.1038/s43856-025-00856-0)

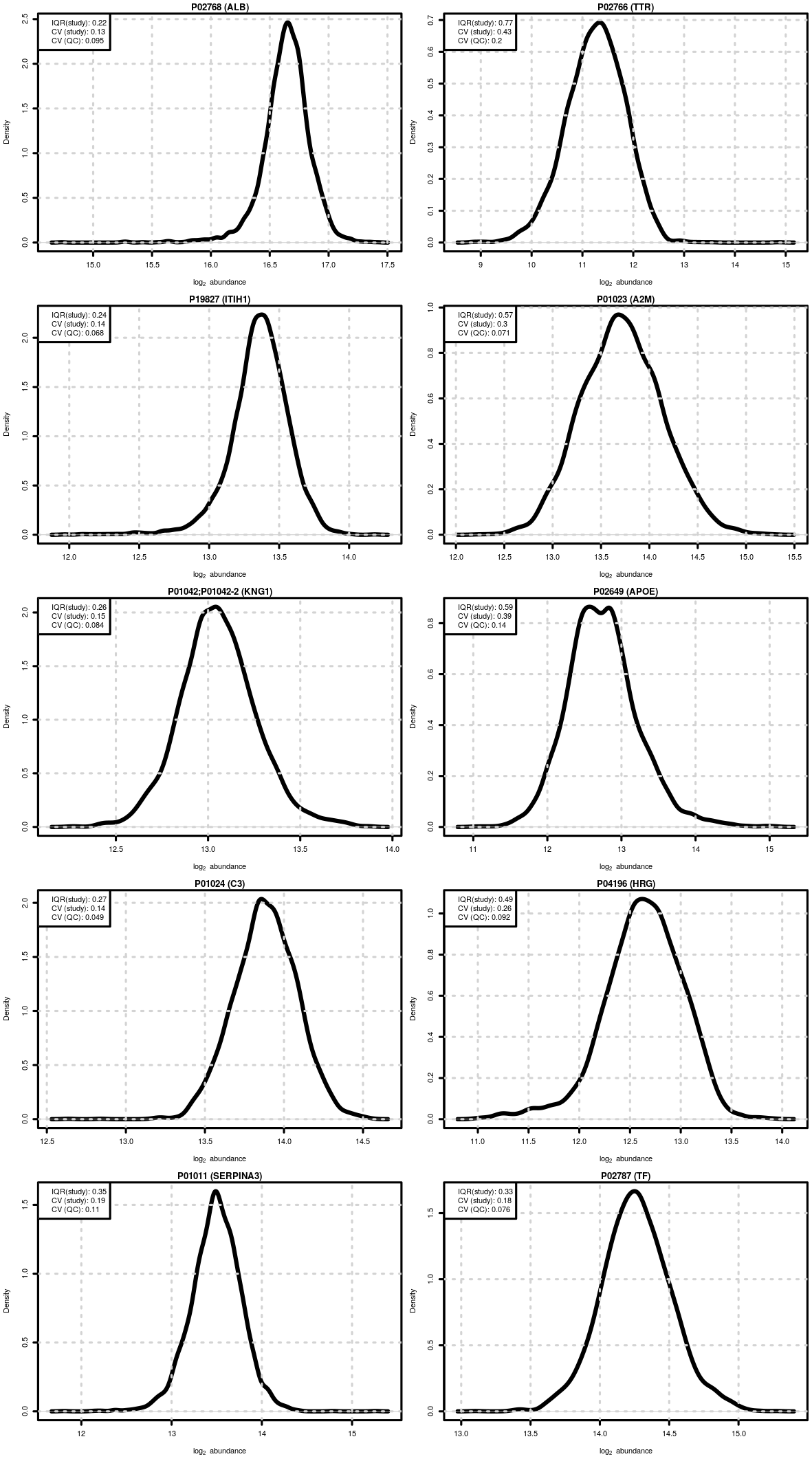

Supplement: Supplementary file 10 — Supplementary Data 7 [file 43856_2025_856_MOESM10_ESM.zip › density-1.png]

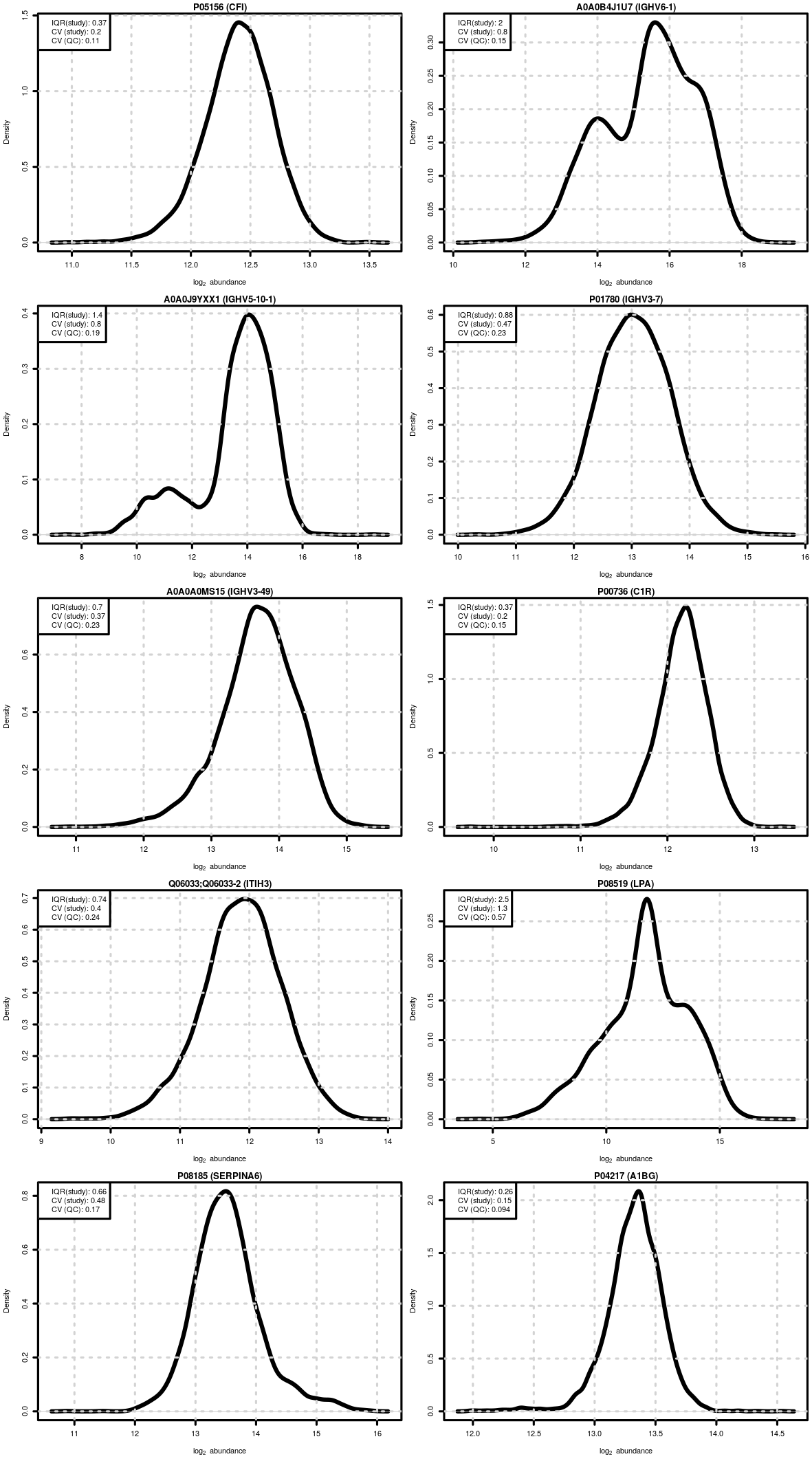

Supplement: Supplementary file 10 — Supplementary Data 7 [file 43856_2025_856_MOESM10_ESM.zip › density-101.png]

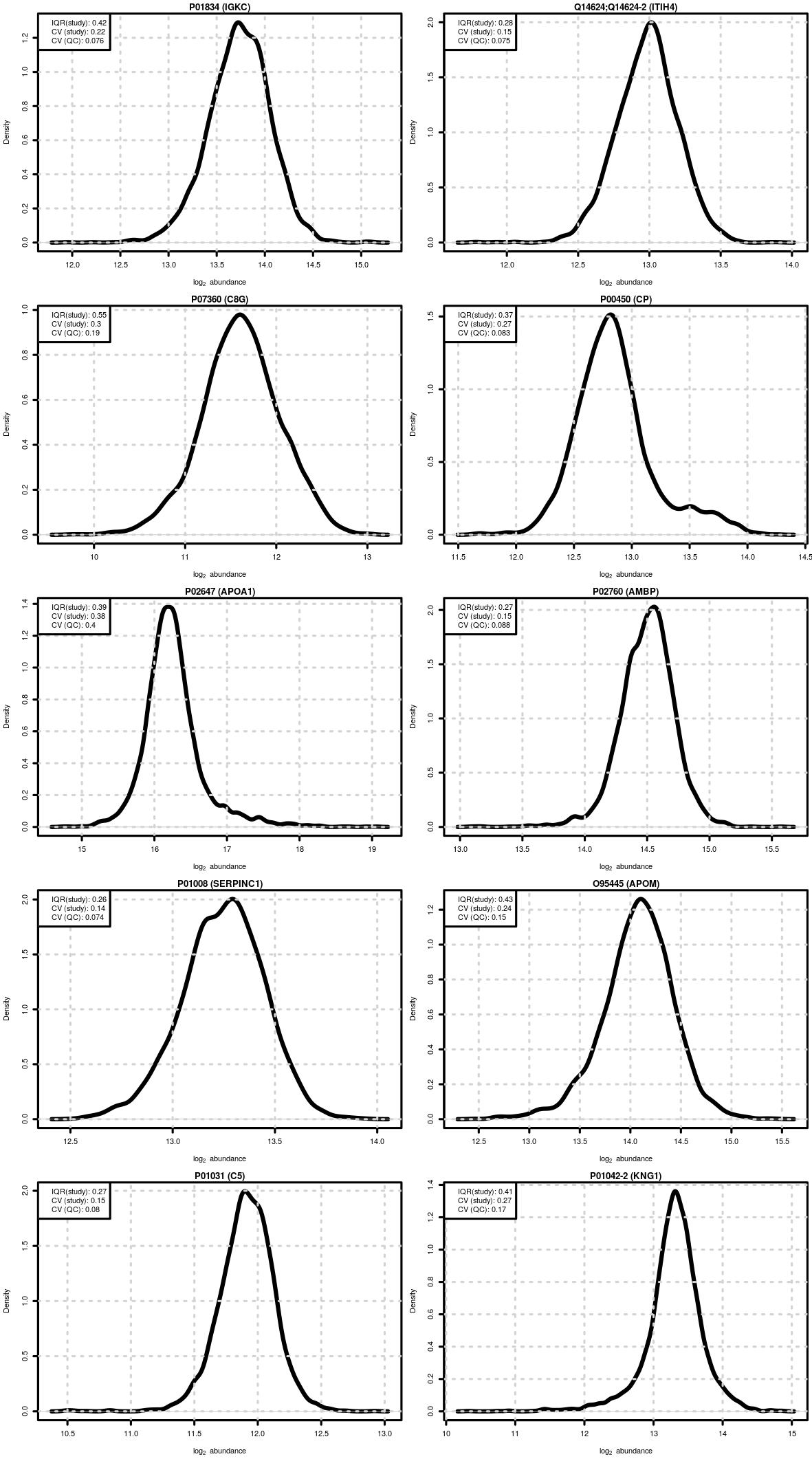

Supplement: Supplementary file 10 — Supplementary Data 7 [file 43856_2025_856_MOESM10_ESM.zip › density-11.png]

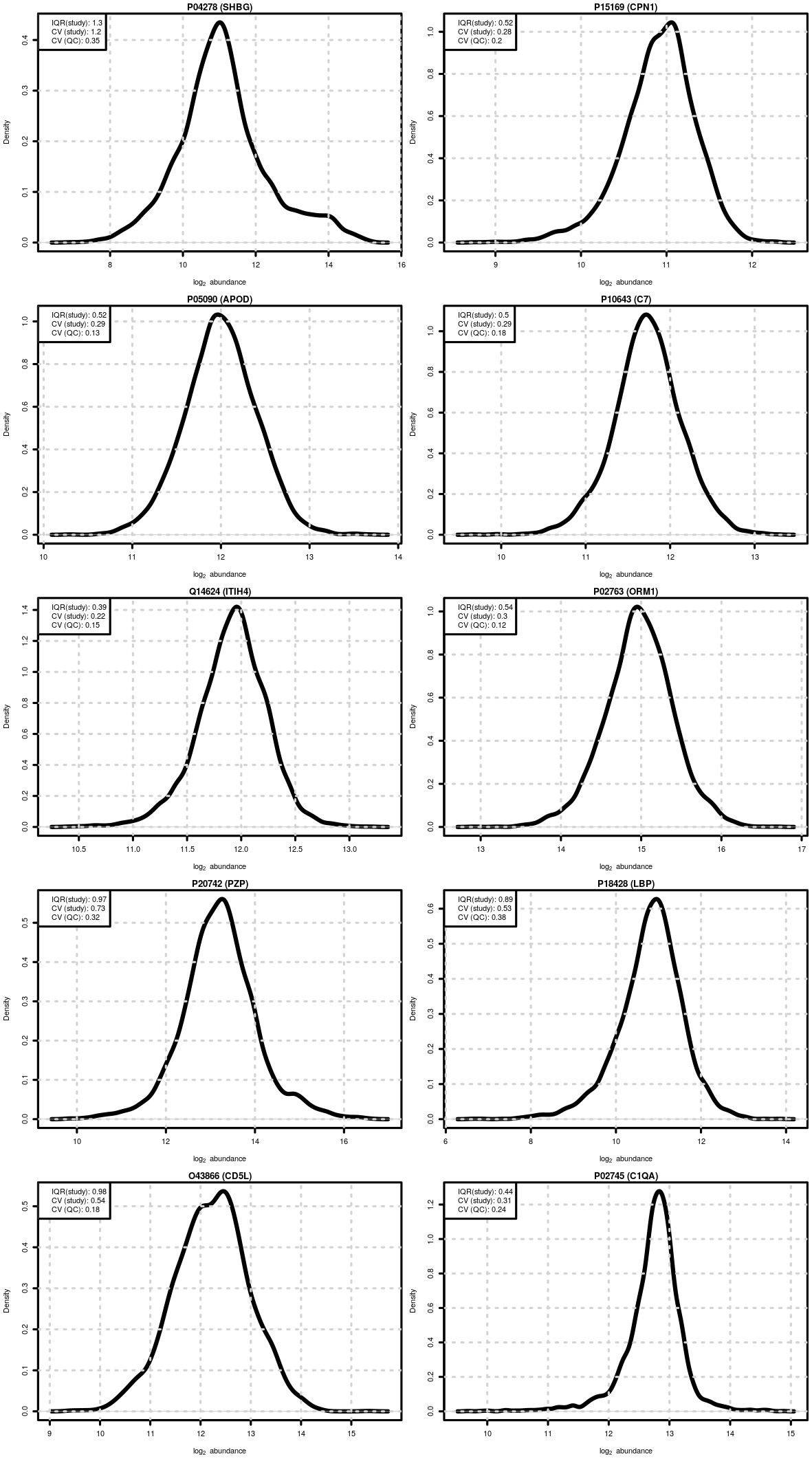

Supplement: Supplementary file 10 — Supplementary Data 7 [file 43856_2025_856_MOESM10_ESM.zip › density-111.png]

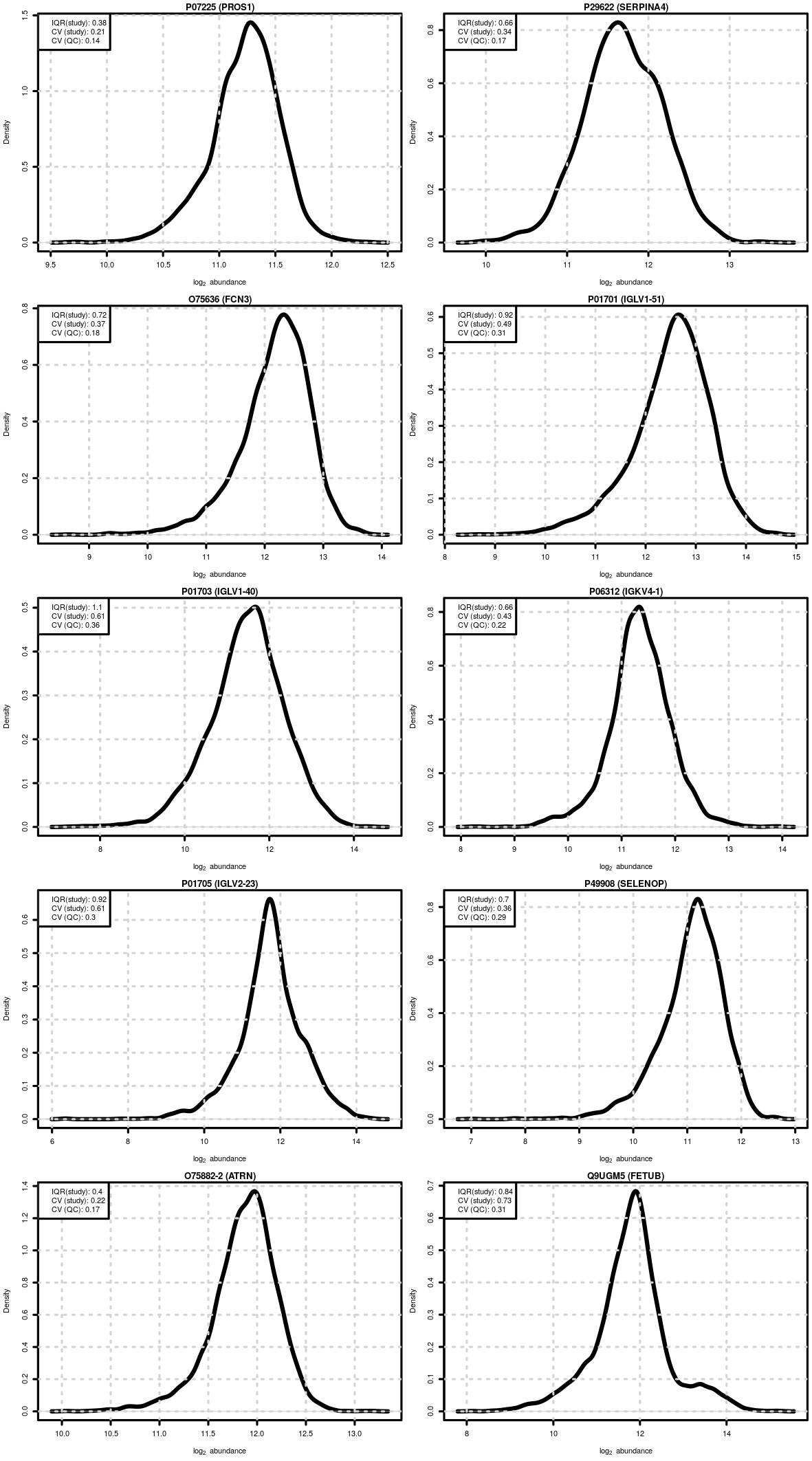

Supplement: Supplementary file 10 — Supplementary Data 7 [file 43856_2025_856_MOESM10_ESM.zip › density-121.png]

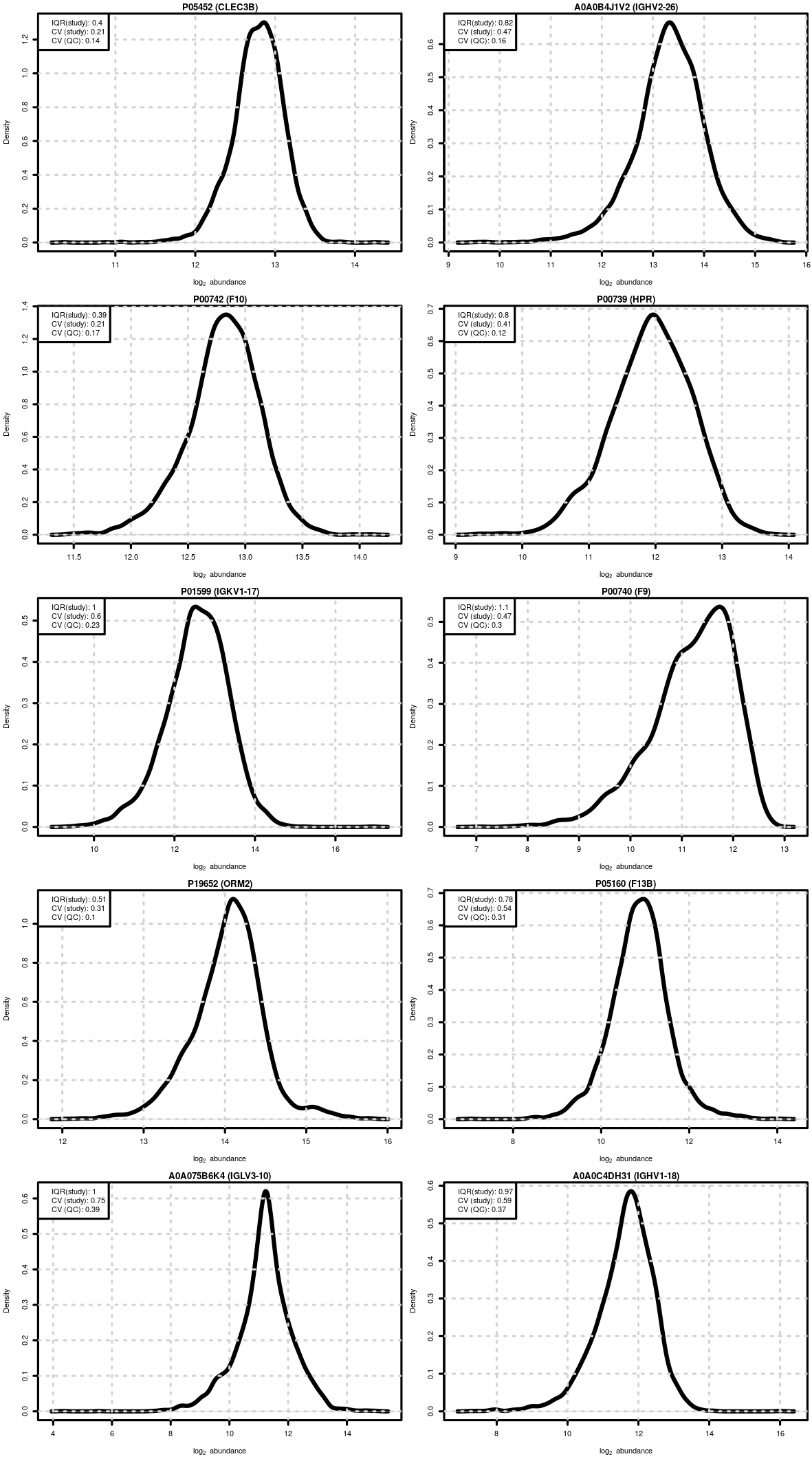

Supplement: Supplementary file 10 — Supplementary Data 7 [file 43856_2025_856_MOESM10_ESM.zip › density-131.png]

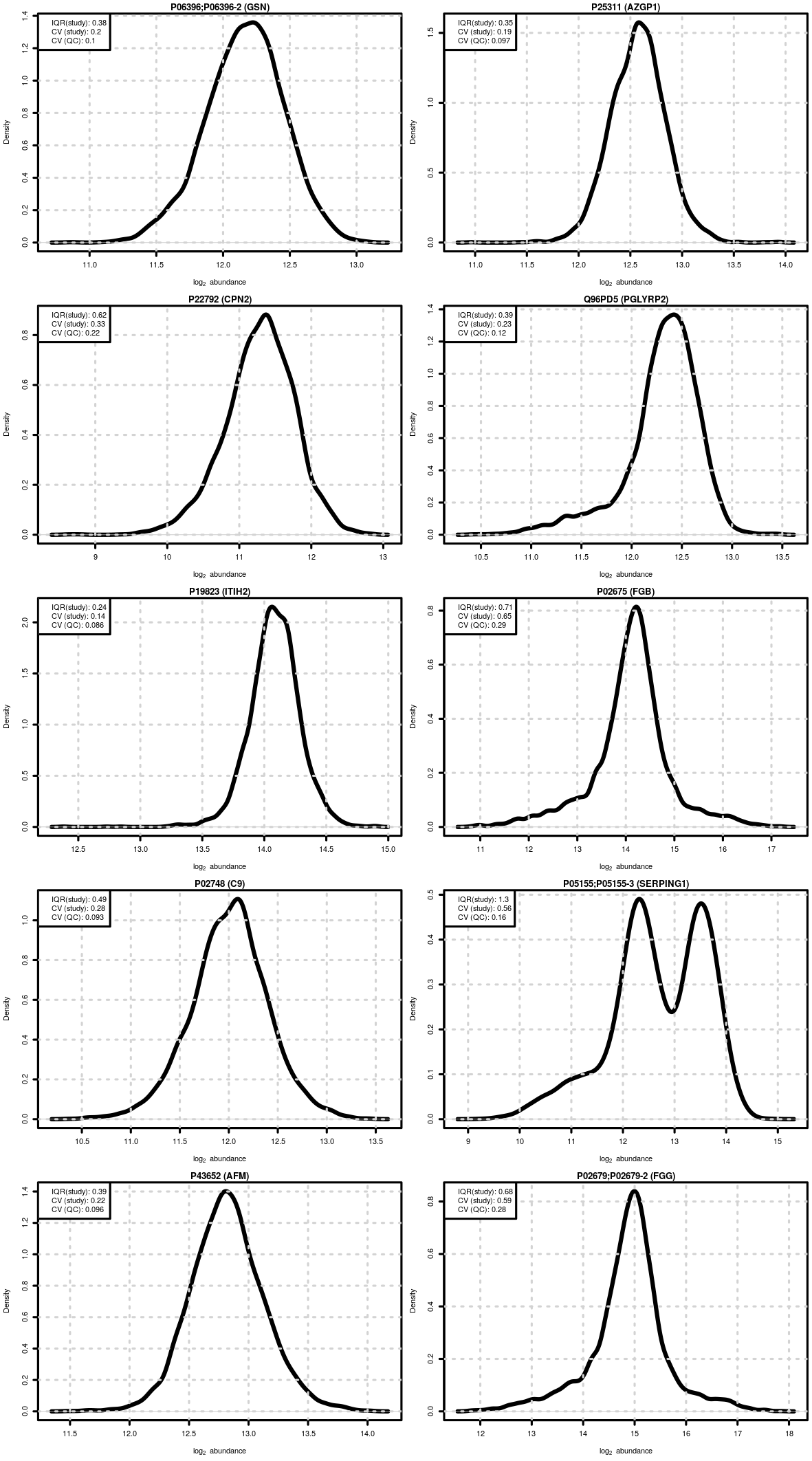

Supplement: Supplementary file 10 — Supplementary Data 7 [file 43856_2025_856_MOESM10_ESM.zip › density-21.png]

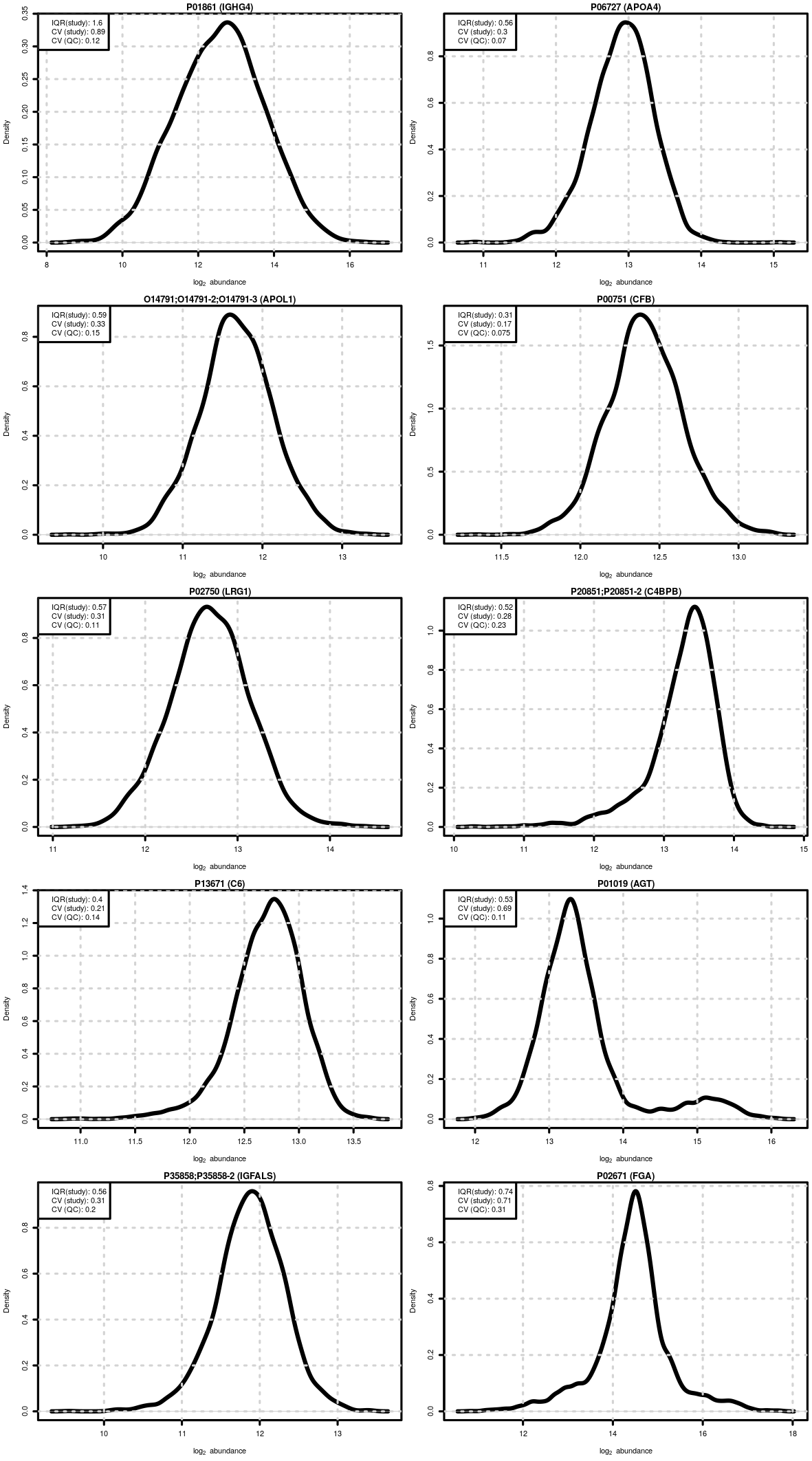

Supplement: Supplementary file 10 — Supplementary Data 7 [file 43856_2025_856_MOESM10_ESM.zip › density-31.png]

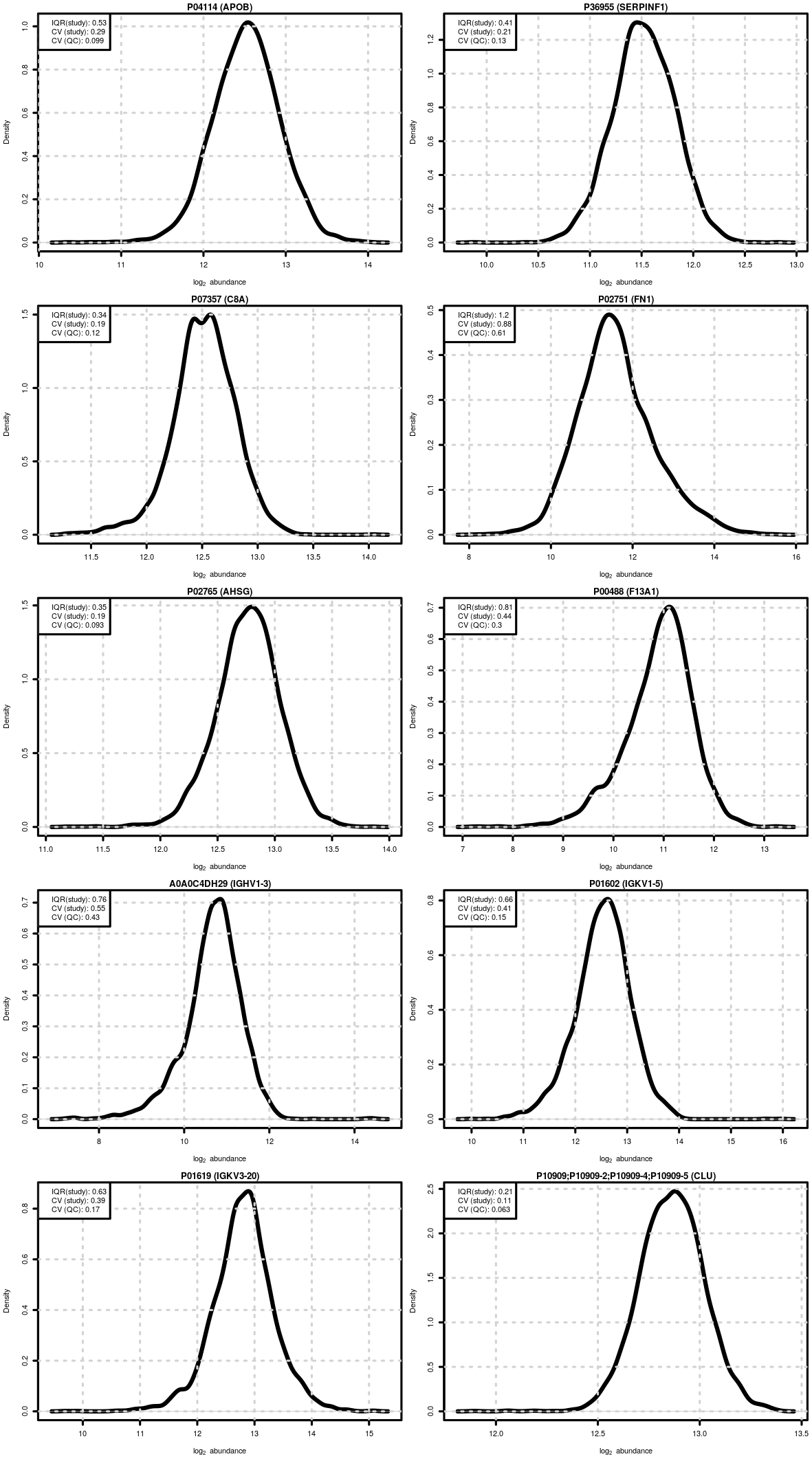

Supplement: Supplementary file 10 — Supplementary Data 7 [file 43856_2025_856_MOESM10_ESM.zip › density-41.png]

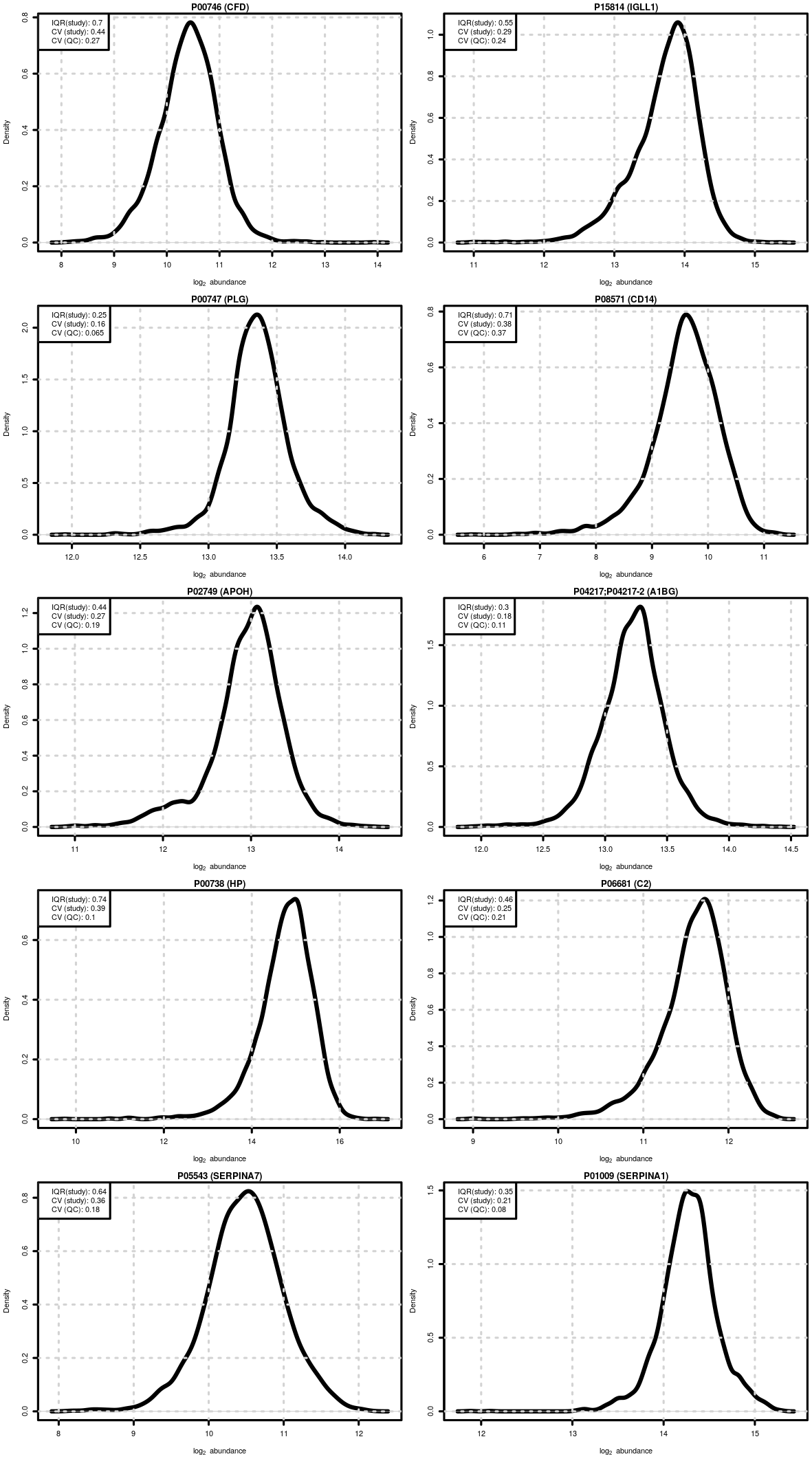

Supplement: Supplementary file 10 — Supplementary Data 7 [file 43856_2025_856_MOESM10_ESM.zip › density-51.png]

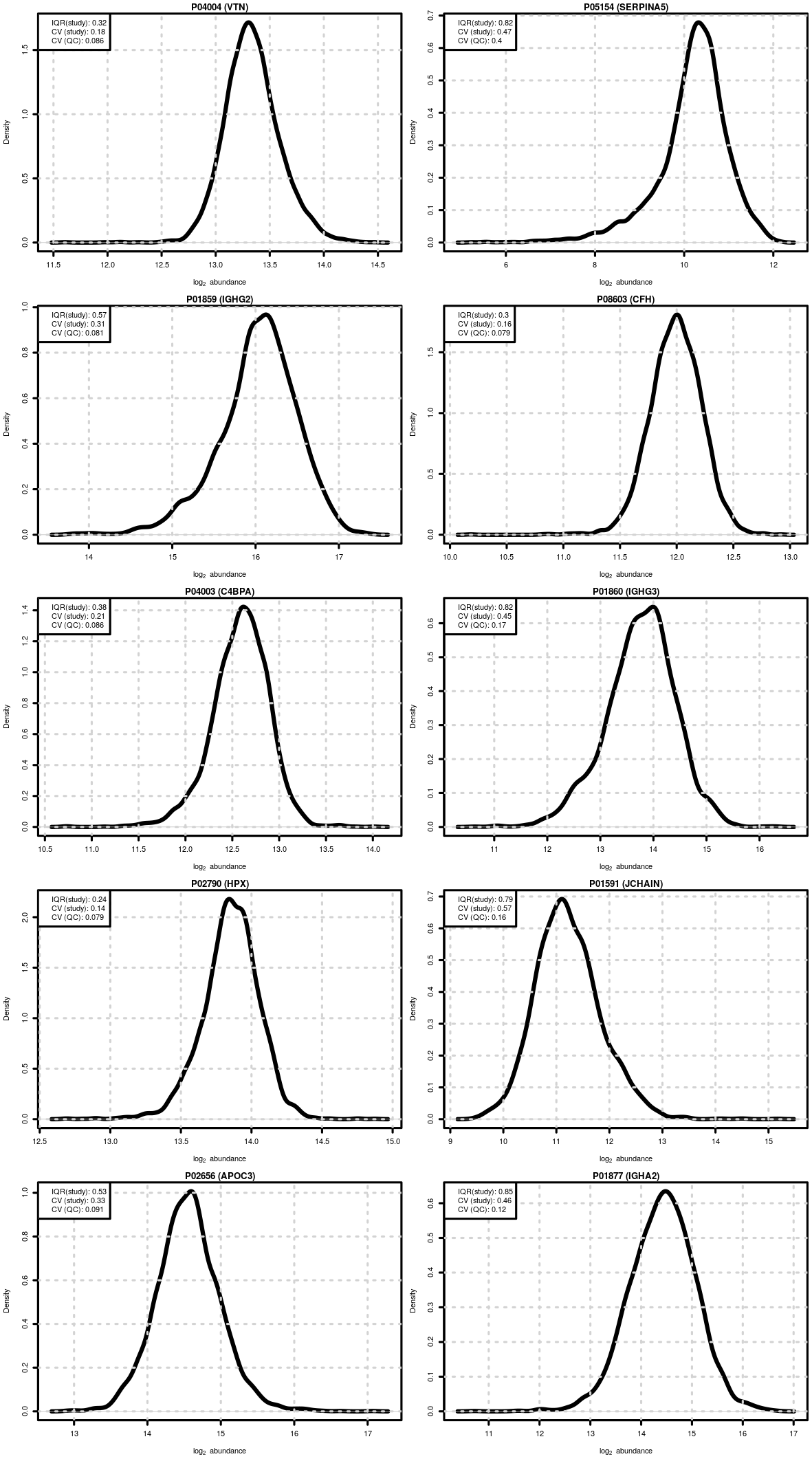

Supplement: Supplementary file 10 — Supplementary Data 7 [file 43856_2025_856_MOESM10_ESM.zip › density-61.png]

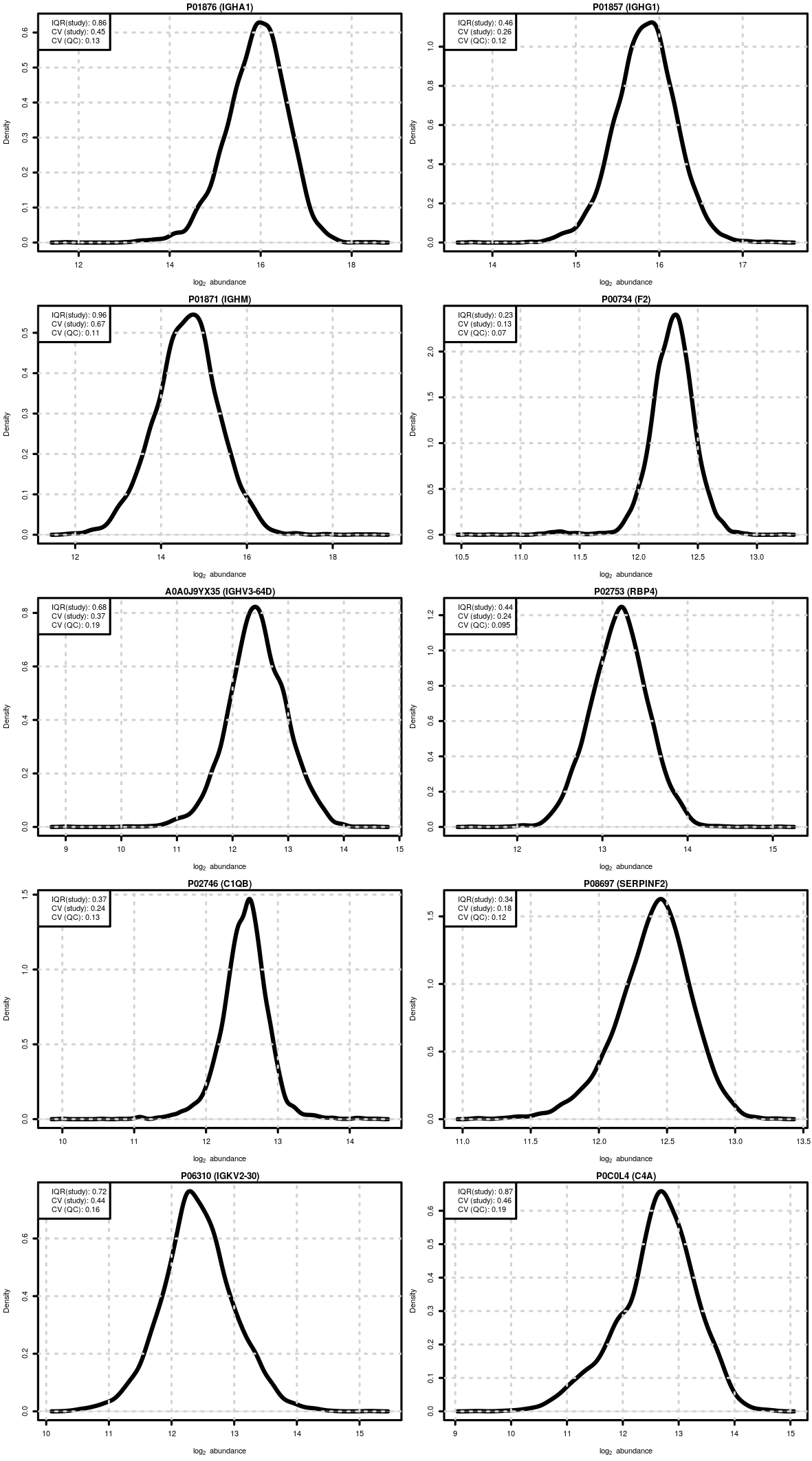

Supplement: Supplementary file 10 — Supplementary Data 7 [file 43856_2025_856_MOESM10_ESM.zip › density-71.png]

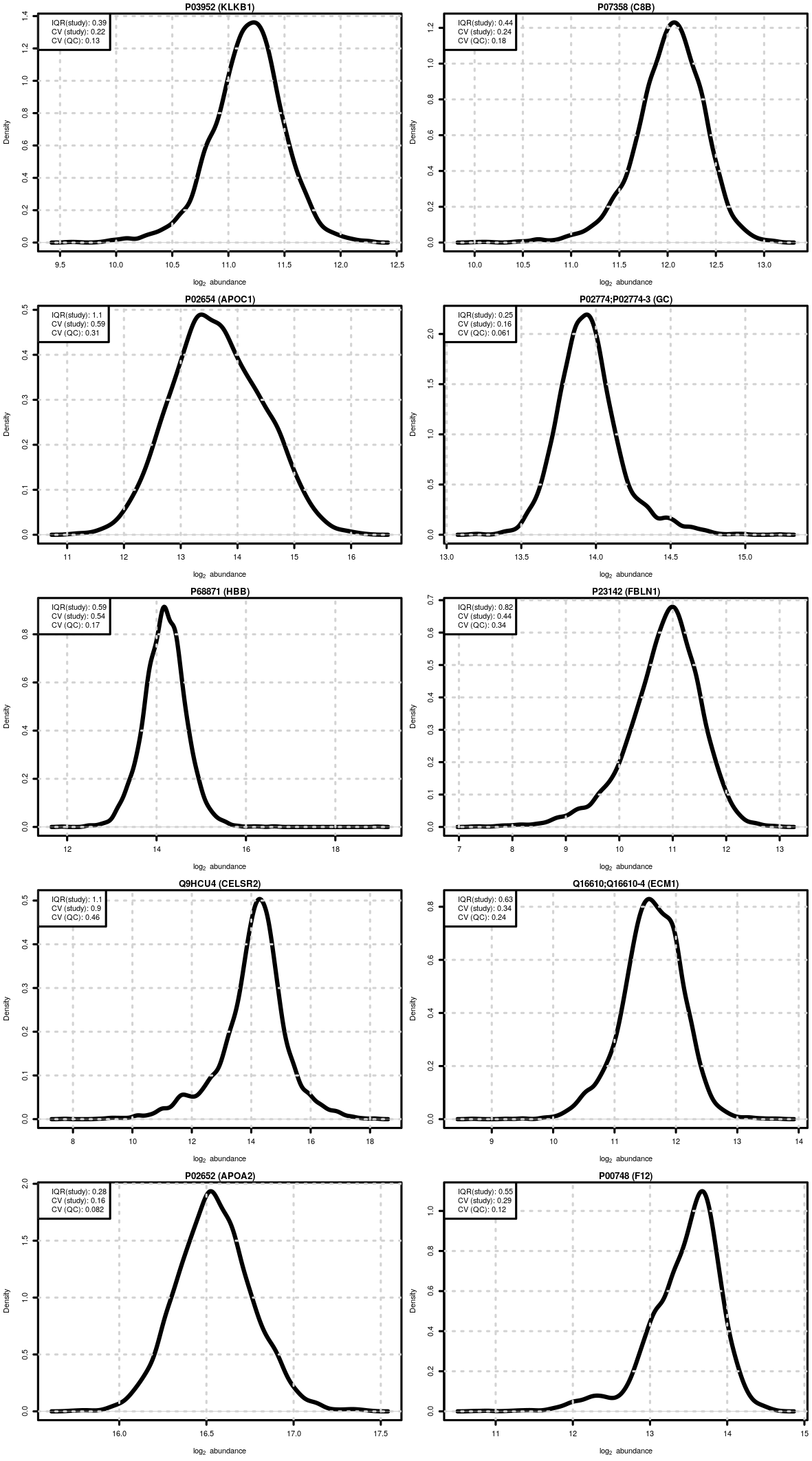

Supplement: Supplementary file 10 — Supplementary Data 7 [file 43856_2025_856_MOESM10_ESM.zip › density-81.png]

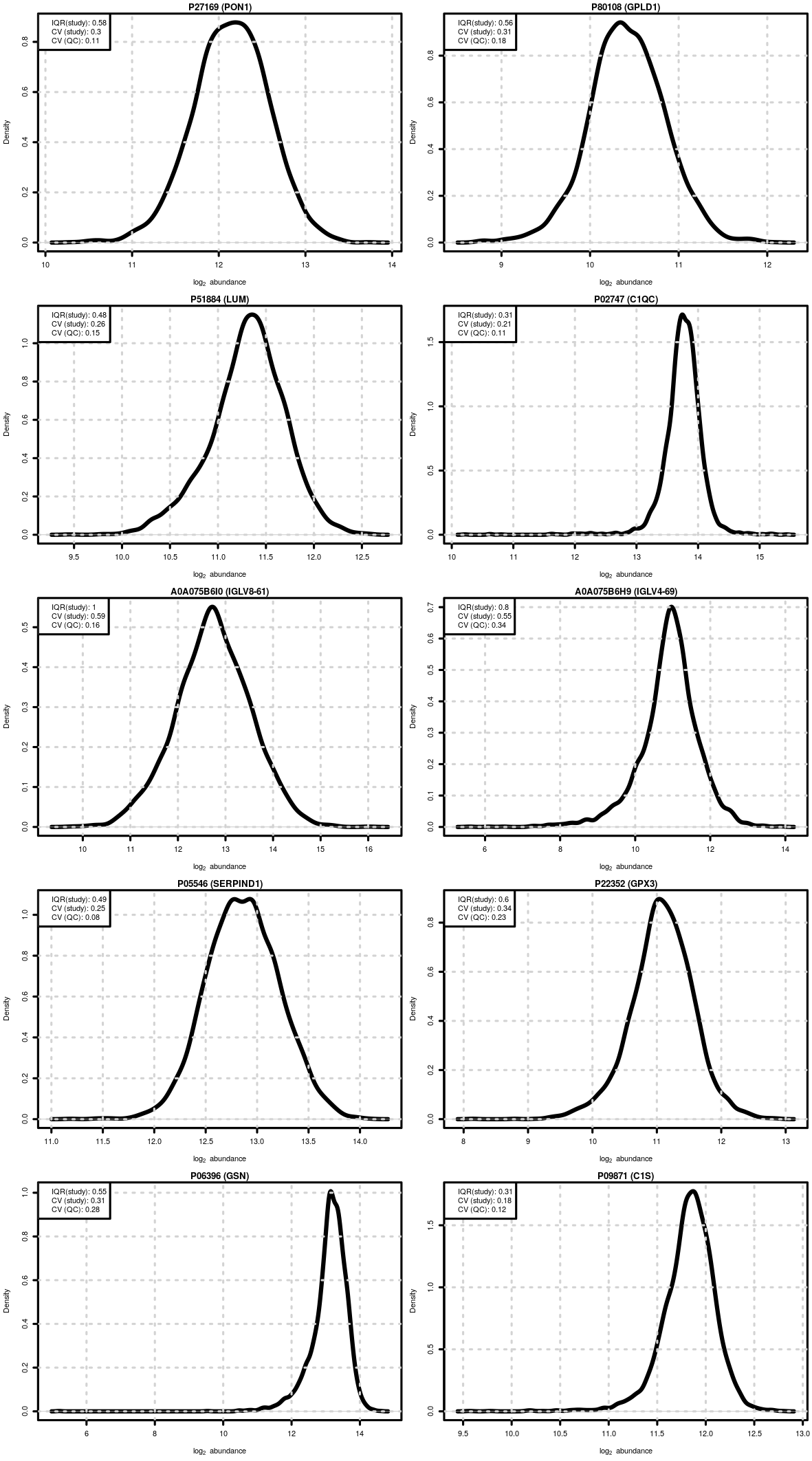

Supplement: Supplementary file 10 — Supplementary Data 7 [file 43856_2025_856_MOESM10_ESM.zip › density-91.png]
