# Supplementary material for: Extensive modulation of the circulating blood proteome by hormonal contraceptive use across two population studies
Source: Commun Med (Lond). 2025 Apr 22;5:131. doi: 10.1038/s43856-025-00856-0 (PMC12015301; doi:10.1038/s43856-025-00856-0)
Supplement: Supplementary file 11 — Reporting Summary [file 43856_2025_856_MOESM11_ESM.pdf]

nature portfolio

Corresponding author(s): Johannes Rainer

Last updated by author(s): Nov 27, 2024

Reporting Summary

Nature Portfolio wishes to improve the reproducibility of the work that we publish. This form provides structure for consistency and transparency in reporting. For further information on Nature Portfolio policies, see our Editorial Policies and the Editorial Policy Checklist.

Statistics

For all statistical analyses, confirm that the following items are present in the figure legend, table legend, main text, or Methods section.

n/a	Confirmed	
☐	☒	The exact sample size (*n*) for each experimental group/condition, given as a discrete number and unit of measurement
☐	☒	A statement on whether measurements were taken from distinct samples or whether the same sample was measured repeatedly
☐	☒	The statistical test(s) used AND whether they are one- or two-sided<br>*Only common tests should be described solely by name; describe more complex techniques in the Methods section.*
☐	☒	A description of all covariates tested
☐	☒	A description of any assumptions or corrections, such as tests of normality and adjustment for multiple comparisons
☐	☒	A full description of the statistical parameters including central tendency (e.g. means) or other basic estimates (e.g. regression coefficient) AND variation (e.g. standard deviation) or associated estimates of uncertainty (e.g. confidence intervals)
☐	☒	For null hypothesis testing, the test statistic (e.g. *F*, *t*, *r*) with confidence intervals, effect sizes, degrees of freedom and *P* value noted<br>*Give P values as exact values whenever suitable.*
☒	☐	For Bayesian analysis, information on the choice of priors and Markov chain Monte Carlo settings
☒	☐	For hierarchical and complex designs, identification of the appropriate level for tests and full reporting of outcomes
☐	☒	Estimates of effect sizes (e.g. Cohen's *d*, Pearson's *r*), indicating how they were calculated

Our web collection on statistics for biologists contains articles on many of the points above.

Software and code

Policy information about availability of computer code

Data collection	Detailed information is provided in the manuscript (Material and Methods section).
Data analysis	Detailed information is provided in the manuscript. Source code (R markdown documents) of the performed analysis is available in a public github repository: https://github.com/EuracBiomedicalResearch/chris_plasma_proteome

For manuscripts utilizing custom algorithms or software that are central to the research but not yet described in published literature, software must be made available to editors and reviewers. We strongly encourage code deposition in a community repository (e.g. GitHub). See the Nature Portfolio guidelines for submitting code & software for further information.

Data

Policy information about availability of data

All manuscripts must include a data availability statement. This statement should provide the following information, where applicable:

- Accession codes, unique identifiers, or web links for publicly available datasets
- A description of any restrictions on data availability
- For clinical datasets or third party data, please ensure that the statement adheres to our policy

The mass spectrometry proteomics data for QC samples, the fasta file used for spectral library annotation and peptide and protein quantities obtained from DIA-NN have been deposited to the ProteomeXchange Consortium (http://proteomecentral.proteomexchange.org) via the PRIDE partner repository. The corresponding PRIDE identifiers for the study pools and quality controls are PXD052861 and PXD052892 respectively.

Individual level data acquired as part of the CHRIS study data can be requested for research purposes by submitting a dedicated request to the CHRIS Access Committee. Please visit https://chrisportal.eurac.edu/ for more information on the process. A similar principle is applied for the BASE-II data. Please contact the scientific coordinator as outlined on https://www.base2.mpg.de/contact.

Human research participants

Policy information about studies involving human research participants and Sex and Gender in Research.

Reporting on sex and gender	We included (self-reported) sex as a covariate in our models to identify plasma proteins significantly associated with sex. The total numbers of women and men included in the analysis are 1,939 and 1,1533, respectively. Due to legal implications and restraints (EU regulations and privacy laws in Italy), sharing of individual level data is not permitted.
Population characteristics	Demographic characteristics are provided in the manuscript. The analyzed study sample consisted of 1,939 female and 1,533 male participants (predominantly healthy individuals). The age range is from 18-90 with the average age for women and men being 45.9 and 46.2, respectively.
Recruitment	The recruitment strategy of the CHRIS population study is detailed in https://pubmed.ncbi.nlm.nih.gov/26541195 . In brief, study participants were recruited by municipality in the Val Venosta, a valley in the northern part of Italy. Invitation letters were sent to all inhabitants aged > 18. Participation to the study was on a voluntary basis and close family members generally participated on the same day/week. Influence of any potential sampling bias on the present analysis is unlikely.
Ethics oversight	CHRIS study: The study was conducted according to the guidelines of the Declaration of Helsinki and approved by the Ethics Committee of the Health Authority of the Autonomous Province of Bolzano (Südtiroler Sanitätsbetrieb/Azienda Sanitaria dell'Alto Adige; protocol No. 21/2011, 19 April 2011). All participants gave written informed consent.<br><br>BASE-II: All participants gave written informed consent. The Ethics Committee of the Charité – Universitätsmedizin Berlin approved the study (approval number EA2/029/09). The study was conducted in accordance with the Declaration of Helsinki and was registered in the German Clinical Trials Registry as DRKS00009277.

Note that full information on the approval of the study protocol must also be provided in the manuscript.

Field-specific reporting

Please select the one below that is the best fit for your research. If you are not sure, read the appropriate sections before making your selection.

☒ Life sciences ☐ Behavioural & social sciences ☐ Ecological, evolutionary & environmental sciences

For a reference copy of the document with all sections, see nature.com/documents/nr-reporting-summary-flat.pdf

Life sciences study design

All studies must disclose on these points even when the disclosure is negative.

Sample size	No sample size calculation was performed. All mass-spectrometry-based proteomics data available for the CHRIS study was used in this analysis and with an n = 3,632 the sample size is expected to be large enough for the present analysis. Results were replicated in proteomics data of an independent cohort. This replication data set included serum proteomics data for 240 women below age of 40.
Data exclusions	Data from pregnant women was not considered in the analysis.
Replication	Results for association with hormonal contraceptives were replicated on new proteomics data of an independent cohort (BASE-II). This replication data set consisted of serum proteomics data for 240 women (age below 40). Results for all investigated traits were in addition compared and validated based on previous results from literature. Quantification of selected proteins were compared against available estimates from clinically certified assays.
Randomization	No randomization was performed as the data was generated from samples of a cross-sectional observational epidemiological study.
Blinding	The data analyzed in the manuscript stems from a cross-sectional observational epidemiological study, thus, no blinding was possible or performed.

Reporting for specific materials, systems and methods

We require information from authors about some types of materials, experimental systems and methods used in many studies. Here, indicate whether each material, system or method listed is relevant to your study. If you are not sure if a list item applies to your research, read the appropriate section before selecting a response.

Materials & experimental systems

n/a	Involved in the study	
☒	☐	Antibodies
☒	☐	Eukaryotic cell lines
☒	☐	Palaeontology and archaeology
☒	☐	Animals and other organisms
☒	☐	Clinical data
☒	☐	Dual use research of concern

Methods

n/a	Involved in the study	
☒	☐	ChIP-seq
☒	☐	Flow cytometry
☒	☐	MRI-based neuroimaging
